# Accurate prediction of subcellular location of apoptosis proteins combining Chou’s PseAAC and PsePSSM based on wavelet denoising

**DOI:** 10.18632/oncotarget.22585

**Published:** 2017-11-21

**Authors:** Bin Yu, Shan Li, Wen-Ying Qiu, Cheng Chen, Rui-Xin Chen, Lei Wang, Ming-Hui Wang, Yan Zhang

**Affiliations:** ^1^ College of Mathematics and Physics, Qingdao University of Science and Technology, Qingdao 266061, China; ^2^ Bioinformatics and Systems Biology Research Center, Qingdao University of Science and Technology, Qingdao 266061, China; ^3^ CAS Key Laboratory of Geospace Environment, Department of Geophysics and Planetary Science, University of Science and Technology of China, Hefei 230026, China; ^4^ Key Laboratory of Eco-Chemical Engineering, Ministry of Education, Laboratory of Inorganic Synthesis and Applied Chemistry, College of Chemistry and Molecular Engineering, Qingdao University of Science and Technology, Qingdao 266042, China; ^5^ College of Electromechanical Engineering, Qingdao University of Science and Technology, Qingdao 266061, China

**Keywords:** apoptosis protein subcellular location, pseudo-amino acid composition, pseudo-position specific scoring matrix, two-dimensional wavelet denoising, support vector machine

## Abstract

Apoptosis proteins subcellular localization information are very important for understanding the mechanism of programmed cell death and the development of drugs. The prediction of subcellular localization of an apoptosis protein is still a challenging task because the prediction of apoptosis proteins subcellular localization can help to understand their function and the role of metabolic processes. In this paper, we propose a novel method for protein subcellular localization prediction. Firstly, the features of the protein sequence are extracted by combining Chou's pseudo amino acid composition (PseAAC) and pseudo-position specific scoring matrix (PsePSSM), then the feature information of the extracted is denoised by two-dimensional (2-D) wavelet denoising. Finally, the optimal feature vectors are input to the SVM classifier to predict subcellular location of apoptosis proteins. Quite promising predictions are obtained using the jackknife test on three widely used datasets and compared with other state-of-the-art methods. The results indicate that the method proposed in this paper can remarkably improve the prediction accuracy of apoptosis protein subcellular localization, which will be a supplementary tool for future proteomics research.

## INTRODUCTION

Protein is involved in various forms of life activities. The living organism's growth, development, reproduction and other life activities are inseparable from the role of the protein. Protein also maintains a highly ordered operation of the protection of the cell system [1]. At the cellular level, proteins function at specific subcellular locations. These locations provide a specific chemical environment and set of interaction partners that are necessary to fulfill the protein's function [2]. Apoptosis is cell physiological death which are closely related to development of organisms, tissue regeneration, immune system regulation and other physiological processes [3]. Studies have shown that apoptosis protein is a protein related to many diseases such as cancer, Alzheimer's disease and so on. It plays an important role in the growth and development of organism [4, 5]. Obtaining information on subcellular location of apoptosis proteins is very helpful to understand the function of apoptosis proteins, the mechanism of cell apoptosis and drug development. Therefore, research of the prediction of subcellular localization of apoptosis proteins has become a hot research topic in proteomics and bioinformatics.

With the life science research has entered into the post-genome era, the accumulated protein sequence data in database of protein increase exponentially. Traditional experimental methods are time-consuming, costly and the repeatability is relatively poor [6]. With the rapid growth of biological data such as nucleic acid and protein sequences, it is far from enough to determine the subcellular location of protein by experimental methods. Therefore, in order to speed up the annotation process of protein structure and function, it is an arduous and challenging task to study how to use machine learning methods to predict protein subcellular localization for researchers. Since the early 1990s, the research of protein subcellular localization prediction has been a hotspot in bioinformatics research. At present, this subject has made great progress [7]. The main research focuses on the following two aspects: (1) Feature extraction of protein sequences. (2) Research and implementation of prediction algorithm.

The feature extraction of protein sequences is currently divided into five categories. (1) N-terminal information prediction method. As early as 1991, Nakai and Kanehisa [8] established a Gram-negative bacterial proteins subcellular localization prediction system by the use of protein N-terminal sequence information. Subsequently, based on the N-terminal sequence information and using the neural network approach, Emanuelsson *et al*. [9] designed the TargetP system to predict protein subcellular location. (2) Protein amino acid composition prediction method. Nakashima *et al*. [10] proposed a prediction method based on the amino acid composition and the frequency of residue pairs. By using the 20 amino acid composition of proteins, they can distinguish intracellular and extracellular proteins, and found the relationship between subcellular localization of protein and its amino acid composition. It is because of their research that many researchers put forward more protein subcellular localization prediction method using amino acid composition [11–13]. For example, Reinhardt *et al*. [11] used amino acid composition as feature information to predict protein subcellular locations in prokaryotes and eukaryotes, and constructed the first artificial neural network prediction system. The method of amino acid composition extraction is convenient, but it does not make full use of the amino acid residues sequence and various physical and chemical properties, so it cannot describe the protein in a comprehensive way. (3) The properties of amino acid residue prediction method. In general, different subcellular regions have different physical and chemical environment. Some researchers take into account the physical and chemical properties of each residue when extracting the protein sequence characteristics. One of the most representative is the pseudo-amino acid composition (PseAAC) proposed by Chou *et al*. [14]. Chou *et al*. [14, 15] defined the PseAAC using the physicochemical properties of amino acids, such as hydrophilicity, hydrophobicity, etc., in combination with the amino acid composition. The predicted subcellular localization accuracy has been significantly improved on the basis of the original. (4) Sequence homology similarity and the protein functional domain prediction method. Homology similarity search is mainly done by means of sequence comparison. Chen *et al*. [16] used the BLAST tool to search for similarity of protein sequences, combined with GO information and sequence characteristics for protein subcellular localization, and finally obtained better predictions. The protein functional domain is a kind of feature extraction method based on annotation information. Chou and Cai [17] predicted protein subcellular localization by fusing PseAAC and functional domain information as feature vectors to obtained better predictive results. Nair and Rost [18] combined the evolutionary information and structural information to predict subcellular localization of proteins, and achieved good predictive results. (5) Multi-feature fusion method for protein sequences. It is difficult to make a big breakthrough in the prediction effect by solely using one certain feature. In recent years, researchers are more inclined to fuse multiple features to characterize the protein sequences, with a view to synthesize the advantages of each sequence coding method to obtain more protein sequences feature information. Gardy *et al*. [19] proposed a coding method PSORT-B, which included amino acid composition, N-terminal sorting signal, and motifs to predict the subcellular localization of Gram-negative proteins. Chen *et al*. [20] proposed a multi-information fusion method to predict subcellular locations of two different types of bacterial proteins by combining the physicochemical properties, auto covariance transformation of the PSSM matrix and GO information, and obtained better results.

Due to the large number of protein sequences and the difficulty of revealing hidden information, so the performance requirement of the prediction algorithm is very high. How to design a predictive algorithm with high throughput, high accuracy and high precision has become another core problem that needs further study. In recent years, pattern recognition methods such as statistics and machine learning have been widely used in prediction algorithms, such as fuzzy K-nearest neighbor (FKNN) [21, 22], neural network [23], hidden Markov model (HMM) [24, 25], Bayesian classifier [26, 27], ensemble classifier [28] and support vector machine (SVM) [29, 30] and so on. Among them, SVM has the advantages of fast computation speed, strong ability of extracting implicit information in training set, excellent generalization performance and so on, which makes SVM as preferred classifier for many researchers.

At present, the prediction of apoptosis protein subcellular localization has made great progress. Zhou and Doctor [31] predicted the subcellular localization of apoptosis proteins for the first time. They used the amino acid composition and covariant discriminate algorithm to predict four kinds of subcellular locations of 98 apoptosis proteins dataset, the overall prediction accuracy achieved 90.8% and 72.5% by re-substitution and jackknife test. Then Bulashevska and Eils [32] used the same dataset with Zhou and Doctor, and by using multiple Bayesian classifier constructed hierarchical ensemble classifier, the overall prediction accuracy was further improved in jackknife test. Zhang *et al*. [33] proposed a new encoding approach with grouped weight for protein sequence and used support vector machine as classifier (named as EBGW_SVM). They constructed a new dataset ZW225 with four apoptosis protein subcellular locations. The overall prediction accuracy of EBGW_SVM achieved 83.1% by jackknife test. Chen and Li [34] proposed a method by combining the increment of diversity with support vector machine (named as ID_SVM). On the new dataset containing 317 apoptosis protein sequences classified into six subcellular locations, and obtained higher prediction accuracy by jackknife test. Based on the CL317 dataset, Ding *et al*. [35] obtained the overall prediction accuracy of 90.9% by using the Fuzzy K-nearest neighbor (FKNN) algorithm. Qiu *et al*. [36] proposed a novel approach by combining discrete wavelet transform with support vector machine (named as DWT_SVM). The overall prediction accuracy of DWT_SVM achieved 97.5%, 87.6% and 88.8% for CL317, ZW225 and ZD98 datasets, respectively by jackknife test. Yu *et al*. [37] used amino acid substitution matrix and auto covariance transformation to extract the sequence features of proteins and construct their feature vectors, and proposed a novel pseudo-amino acid model to predict subcellular localization of apoptosis proteins. For ZW225 and CL317, the higher prediction overall accuracy was 87.1% and 90.0%, respectively by jackknife test. Liu *et al*. [38] proposed a method for the prediction of subcellular localization of apoptosis proteins based on tri-gram encoding of position-specific scoring matrices and incorporating evolution information of proteins. For the ZW225, CL317 and ZD98 datasets, the higher accuracy of prediction was 97.8%, 95.9% and 96.9%, respectively by jackknife test. Liang *et al*. [39] proposed a new feature extraction method to predict apoptosis protein subcellular localization by fusing Geary autocorrelation function and detrended cross-correlation coefficient (DCCA) based on PSSM. For three benchmark datasets ZD98, ZW225 and CL317, the overall prediction accuracy achieved 91.8%, 84.4% and 89.0%, respectively. Dai *et al*. [40] and Xiang *et al*. [41] proposed an information extraction algorithm based on the golden ratio segmentation of protein sequences. The PSSM matrix of apoptosis protein sequence was split into several different sub-matrixes by golden ratio, and the evolution of the statistical sub-matrices information. It is found that the prediction model based on the composition information, position information and evolution information can significantly improve the subcellular localization prediction accuracy of apoptosis proteins.

In this paper, we propose a novel method for predicting the subcellular localization of apoptosis proteins, called PseAAC-PsePSSM-WD. Firstly, the features are extracted from apoptosis protein sequences by combining Chou's PseAAC and PsePSSM algorithms. Then, the feature vectors of the extracted proteins are denoised by two-dimensional wavelet, which make the features of each class of proteins are more prominent after wavelet denoising. Finally, the optimal feature vectors after wavelet denoising are input to the SVM classifier for prediction. By jackknife test, different effects on the results are compared due to choosing different λ values, ξ values (where the λ values and ξ the order information of protein amino acid sequences), wavelet functions, different wavelet decomposition scales, different feature extraction algorithm, different kernel functions and classifiers. Through the comparative analysis, the optimal parameters of the model are determined, and the subcellular localization prediction model of apoptosis proteins are established. On the three benchmark datasets CL317, ZW225 and ZD98, obtain the highest overall prediction accuracy of 99.37%, 100% and 98.98%, respectively by the most rigorous jackknife test. According to the comparision with other existing methods, the experimental results show that our method can remarkably improve the prediction accuracy of protein subcellular localization.

## RESULTS AND DISCUSSION

### Selection of optimal parameter λ and *ξ*

The selection of parameters is very important for a prediction system. How to extract effective feature information from protein sequence is the key to success of protein subcellular localization prediction model. In order to better discover the merits of the characteristic parameters, it is usually necessary to make a comparison. The apoptosis protein datasets CL317 and ZW225 are used as the research object, and the optimal parameters of the predicted model are selected.

In current study, we use PseAAC and PsePSSM algorithm to carry out feature extraction on protein sequences. In the process of feature extraction, the selection of values λ and *ξ* play an important role on the construction of the model. Both value λ and *ξ* represent sequence-order information of the amino acid residues in the protein sequence. If the value λ and *ξ* are set too large, it will make the dimension of feature vector of protein sequence too high, bring more redundant information, thus affecting the prediction results. If the value λ and *ξ* are set too small, the sequence information contained in the feature vectors will be very little, and the features of the protein sequence on the apoptosis datasets cannot be extracted completely. To find the optimal value λ in the model, set the values *λ,* from 0 to 49 in turn. For the different values of *λ*, the SVM is used to classify apoptosis datasets CL317 and ZW225, respectively. SVM uses the linear kernel function and the results are tested by jackknife method. The overall prediction accuracy of each class of the apoptosis datasets are shown in Tables 1 and 2. Similarly, to find the optimal value *ξ* in the model, set the values *ξ* from 0 to 10 in turn. For the different values of *ξ*, the SVM is used to classify apoptosis datasets CL317 and ZW225, respectively. SVM uses the linear kernel function and the results are tested by jackknife method. The overall prediction accuracy of each class of the apoptosis datasets are shown in Tables 3 and 4.

**Table 1:** Prediction results of subcellular localization of the CL317 dataset by selecting different *λ* values

Locations	*λ*										
	Jackknife test (%)										
	0	5	10	15	20	25	30	35	40	45	49
Cy	80.36	79.46	86.61	88.39	86.61	84.82	87.50	85.71	83.03	83.93	83.93
Me	74.55	80.00	85.45	90.91	89.09	89.09	87.27	83.64	87.27	87.27	85.45
Mi	58.82	67.65	70.59	73.53	64.71	73.53	61.76	73.53	67.65	61.76	67.65
Se	29.41	47.06	58.82	41.18	47.06	47.06	58.82	64.71	70.59	58.82	58.82
Nu	57.69	76.92	71.15	78.85	71.15	80.76	71.15	75.00	75.00	75.00	75.00
En	89.36	93.62	93.62	95.74	95.74	97.87	95.74	95.74	95.74	93.62	93.62
OA	71.92	78.23	81.70	84.23	81.39	83.60	81.70	82.65	82.02	80.76	81.07

**Table 2:** Prediction results of subcellular localization of the ZW225 dataset by selecting different *λ* values

Locations	*λ*										
	Jackknife test (%)										
	0	5	10	15	20	25	30	35	40	45	49
Cy	81.43	85.71	80.00	85.71	84.29	80.00	80.00	81.43	78.57	81.43	78.57
Me	83.15	86.52	91.01	87.64	91.01	88.76	88.76	91.01	88.76	87.64	85.39
Mi	52.00	52.00	56.00	64.00	56.00	60.00	56.00	56.00	56.00	60.00	56.00
Nu	70.73	65.85	56.10	63.41	60.98	65.85	68.29	75.61	70.73	68.29	75.61
OA	76.89	78.67	77.33	80.00	79.56	78.67	78.67	81.33	78.67	79.11	78.22

**Table 3:** Prediction results of subcellular localization of the CL317 dataset by selecting different *ξ* values

Locations	*ξ*										
	Jackknife test (%)										
	0	1	2	3	4	5	6	7	8	9	10
Cy	83.04	83.93	87.50	87.50	87.50	89.29	91.96	91.96	91.96	91.07	91.96
Me	78.18	81.82	90.91	90.91	89.09	90.91	90.91	89.09	89.09	90.91	89.09
Mi	50.00	55.88	70.59	73.53	82.35	82.35	82.35	85.29	85.29	85.29	88.24
Se	88.24	82.35	82.35	88.24	82.35	82.35	82.35	82.35	82.35	82.35	82.35
Nu	67.31	61.54	78.85	80.77	86.54	90.38	90.38	88.46	88.46	90.38	88.46
En	87.23	91.49	93.62	93.62	93.62	93.62	93.62	95.74	97.87	97.87	97.87
OA	76.97	77.92	85.49	86.44	87.70	89.27	90.22	90.22	90.54	90.85	90.85

**Table 4:** Prediction results of subcellular localization of the ZW225 dataset by selecting different *ξ* values

Locations	*ξ*										
	Jackknife test (%)										
	0	1	2	3	4	5	6	7	8	9	10
Cy	81.43	80.00	82.86	84.29	80.00	81.43	85.71	84.29	84.29	84.29	84.29
Me	82.02	85.39	89.89	91.01	91.01	91.01	91.01	92.13	91.01	91.01	91.01
Mi	36.00	60.00	68.00	68.00	72.00	72.00	72.00	76.00	72.00	72.00	72.00
Nu	63.41	65.85	70.73	75.61	80.49	78.05	82.93	82.93	82.93	82.93	85.37
OA	73.33	77.33	81.78	83.56	83.56	83.56	85.78	86.22	85.33	85.33	85.78

As can be seen from Table 1, different prediction results will be got by changing the value of *λ*. With the constant change of *λ* value, the predictive accuracy of each class of the proteins and the overall accuracy of the model are also constantly changing. For the CL317 dataset, when *λ* = 15, the highest prediction accuracy of cytoplasmic proteins reach 88.39%. When *λ* = 25, endoplasmic proteins get the highest prediction accuracy is 97.87%, 2.13% higher than when *λ* = 15. Similarly, when *λ* = 25, the highest prediction accuracy of membrane proteins, mitochondrial proteins and nuclear proteins reach 89.09%, 73.53% and 80.76%, respectively. Considering the influence of different *λ* value on the prediction results and the analysis of overall prediction accuracy of CL317 dataset, the highest overall prediction accuracy is 84.23% when *λ* = 15.

As can be seen from Table 2, for the ZW225 dataset, the cytoplasmic proteins reach the highest prediction accuracy when *λ* = 5 and *λ* = 15, which are 85.71%. When *λ* = 10, *λ* = 20 and *λ* = 35, membrane proteins reach the highest prediction accuracy, which are 91.01%, 3.37% higher than when *λ* = 15. For the mitochondrial proteins, the highest prediction accuracy is 64.00% when *λ* = 15, and the prediction accuracy is significantly lower than that of other types of protein prediction accuracy. It is possibly because the number of mitochondrial proteins in the ZW225 dataset is 25, the small amount of data affect the model construction effect. When *λ* = 35 and *λ* = 49, nuclear proteins reach the highest prediction accuracy of 75.61%. Through the analysis of the prediction results of ZW225 dataset, the overall highest prediction accuracy of the model is 81.33% when *λ* = 35.

**Figure 1:**
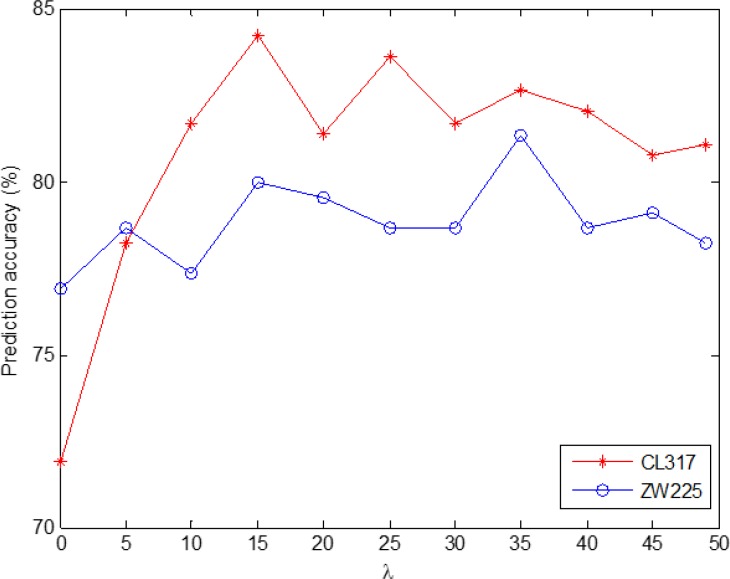
Effect of selecting different values of *λ* on the prediction results of subcellular localization for CL317 and ZW225 datasets

In order to more intuitively find the optimal *λ* value, Figure 1 is the change of overall prediction accuracy rate of CL317 and ZW225 datasets when choose different *λ* values. As can be seen from Figure 1, with the *λ* value of the change, the prediction accuracy of the two datasets are also changing. In addition, *λ* values are different for the highest accuracy of two datasets. In order to unify the parameters of the model, we choose optimal value *λ* = 15. Therefore, the PseAAC algorithm is used to extract the protein sequence, and each protein sequence generates a 20 *+ λ* = 35 dimension feature vector.

As can be seen from Table 3, the constant change of *ξ* value will have different influence on the prediction accuracy of each class of proteins in CL317 dataset. For cytoplasmic proteins, the highest prediction accuracy is 91.96% when *ξ* values are 6, 7, 8 and 10, respectively. For endoplasmic proteins, the highest prediction accuracy of the protein is 97.87% when *ξ* values are 8, 9 and 10, respectively. For membrane proteins, the highest prediction accuracy of the protein is 90.91% when *ξ* values are 2, 3, 5, 6 and 9, respectively. For mitochondrial proteins, the highest prediction accuracy of the protein is 88.24% when *ξ* = 10. For nuclear proteins, the highest predictive accuracy of the protein is 90.38% when *ξ* values are 5, 6 and 9. For secreted proteins, the highest prediction accuracy of the protein is 88.24% when *ξ* values are 0 and 3. The highest overall prediction accuracy of dataset CL317 is 90.85% when *ξ* value is 9 or 10.

As can be seen from Table 4, the constant change of *ξ* value will have different effect on prediction accuracy of each class of protein in ZW225 dataset. For cytoplasmic proteins, the highest prediction accuracy is 85.71% when *ξ* = 6. For membrane proteins, the highest predictive accuracy of the protein is 92.13% when *ξ* = 7. For mitochondrial proteins, the highest prediction accuracy of the protein is 76.00% when *ξ* = 7. For nuclear proteins, the highest prediction accuracy of the protein is 85.37% when *ξ* = 10. The highest overall prediction accuracy of dataset ZW225 is 86.22% when *ξ* = 7.

**Figure 2:**
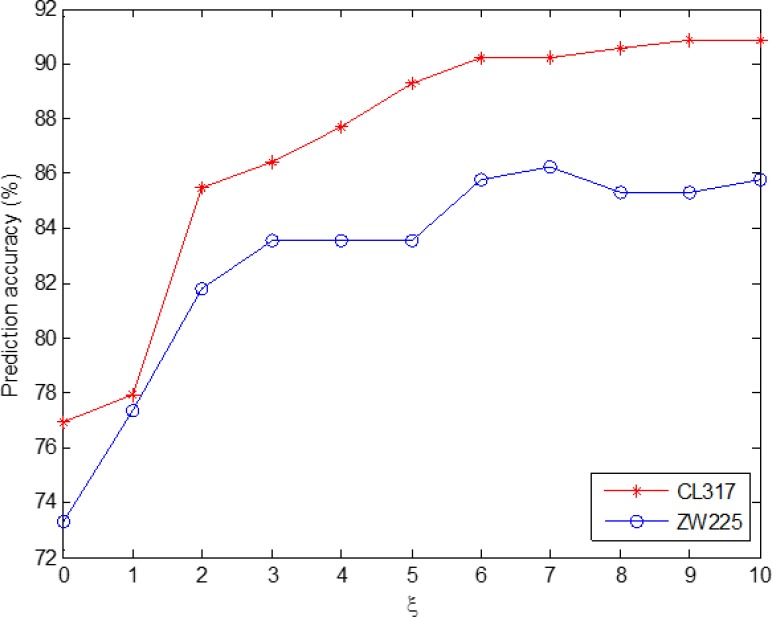
Effect of selecting different values of *ξ* on the prediction results of subcellular localization for CL317 and ZW225 datasets

Since the two apoptosis protein datasets CL317 and ZW225 are selected in this paper, in order to make the selection of model parameters consistent, the two datasets are analyzed integrally. Figure 2 shows the change in overall prediction accuracy when the two datasets choose different *ξ* values. We choose optimal parameter *ξ* = 10 of the model, the PsePSSM algorithm can be used to extract each protein sequence to obtain 20 + 20 × *ξ* = 220 dimension feature vector.

### Selection of wavelet function and optimal decomposition scale

In order to achieve the ideal prediction accuracy of subcellular localization of apoptosis proteins, we fused the PseAAC and PsePSSM algorithms to extract the characteristics of protein sequences. Each protein sequence in the dataset generates (20 + *λ*) + (20 + 20 × *ξ*) = 35 + 220 = 255 dimension feature vector, and wavelet denoising method is used to extract information furtherly. Since the wavelet basis function produces different wavelet families, each family has its own characteristics, that is to say, different wavelet family has different processing ability for different data. If the characteristics of the wavelet function can better match message structure of the signal information, the better feature information can be extracted from the sequences [42]. In order to obtain the best performance of the model in the data processing, the selection of the wavelet function is very important for the construction of the model. In addition, in the analysis of protein sequences, different decomposition scales will get different prediction results. Decomposing a longer sequence with too low a decomposition scale level would omit much detailed information, and decomposing a shorter sequence with too high a decomposition scale would inevitably introduce redundant information [43]. In order to extract the protein sequences feature information of apoptosis proteins more effectively, this paper examines the effects of different wavelet functions and different decomposition scales on the prediction model.

When 2-D denoising method is used to carry out feature extraction on datasets, there are two different methods for threshold selection, the default threshold, and the threshold gained by Birge-Massart strategy. In the process of noise reduction, using the default threshold will result in a uniform global threshold, whereas thresholds obtained using the Birge-Massart strategy will generate three different thresholds in horizontal, vertical and diagonal. There are two methods to get the default threshold, one is to use the wavelet function to obtain the default threshold, and the other is to use the wavelet packet to obtain the default threshold. For the apoptosis protein datasets CL317 and ZW225, after several experiments, it is found that using the wavelet function to obtain the default threshold can obtain the highest overall prediction accuracy on the two datasets at different decomposition scales and different wavelet functions (The specific results see Supplementary Tables 1 and 2). In the following discussion, we use the wavelet function to obtain the default threshold for 2-D wavelet denoising. The PseAAC and PsePSSM algorithms are used to extract the features of the protein sequence. When PseAAC is used to extract feature information, *λ* = 15 is chosen. When PsePSSM is used to extract feature information, *ξ* = 10 is chosen. The linear kernel function with SVM is used to classify and the results is validated by jackknife test. The prediction results of subcellular localization of two apoptosis protein datasets under different wavelet functions and different decomposition scales are obtained, as shown in Table 5.

**Table 5:** Prediction results of subcellular localization in the datasets CL317 and ZW225 under different wavelet functions and different decomposition scales

Datasets	Functions										
	Jackknife test (%)										
		db1	db4	db8	sym3	sym7	coif2	coif4	bior1.1	bior2.4	bior3.3
CL317	3	99.05	98.11	99.05	98.74	98.11	98.11	97.48	99.05	98.42	98.74
	4	98.74	97.48	99.37	98.11	97.79	98.74	98.42	98.74	98.42	98.74
	5	98.74	97.79	99.05	98.74	98.42	98.11	98.42	98.74	98.42	98.74
ZW225	3	98.67	98.67	100	99.11	99.11	99.56	99.56	98.67	98.22	99.11
	4	98.22	98.67	100	98.22	98.67	99.11	99.11	98.22	97.78	99.11
	5	97.78	98.22	99.56	99.11	99.11	98.67	99.56	97.78	97.78	98.67

**Figure 3:**
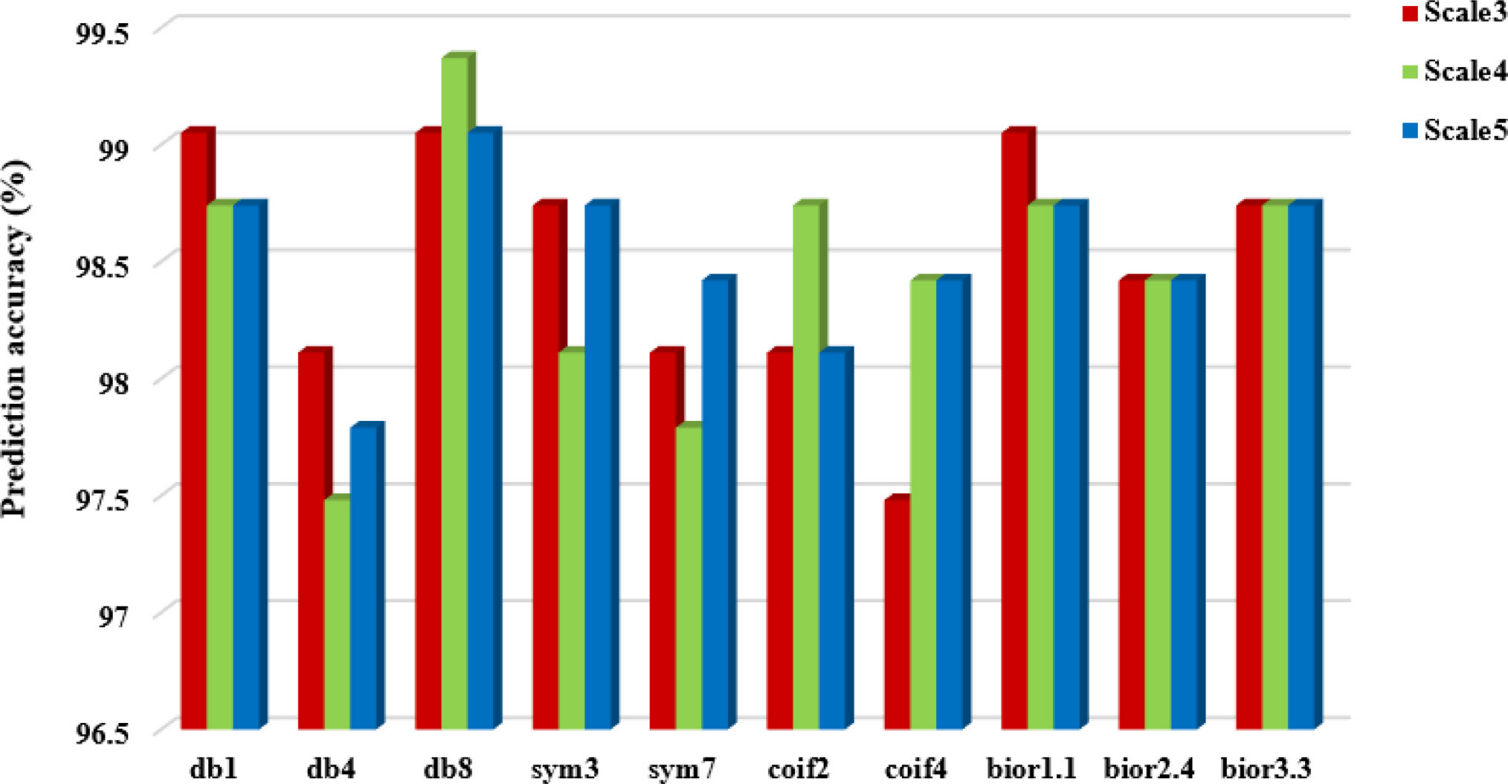
Prediction performance of dataset CL317 under different wavelet functions and different decomposition scales

**Figure 4:**
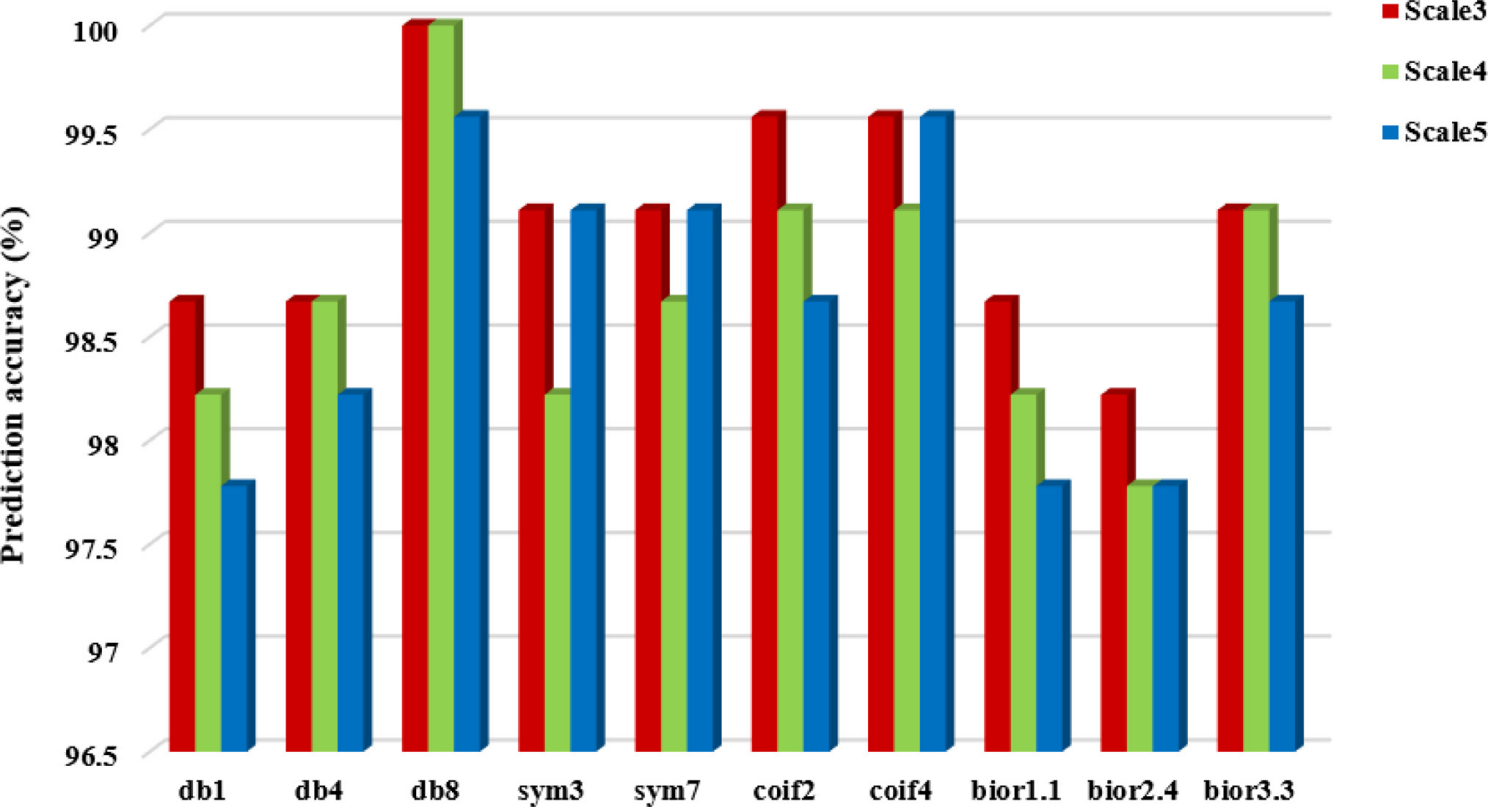
Prediction performance of dataset ZW225 under different wavelet functions and different decomposition scales

It can be seen from Table 5 that choosing different wavelet functions and different decomposition scales can affect the overall prediction accuracy of the model. For the CL317 dataset, when the decomposition scale is 3, the db1, db8 and bior1.1 wavelet function are selected respectively, and the highest overall prediction accuracy of subcellular localization is 99.05%. When the decomposition scale is 4, the highest prediction accuracy is 99.37%, and the corresponding wavelet function is db8. When the decomposition scale is 5, the db8 wavelet is used to obtain the highest prediction accuracy of 99.05%. Therefore, with the difference of wavelet decomposition scales, the prediction model also changes the wavelet function corresponding to the highest prediction accuracy of CL317 dataset. It is worth noting that the db8 wavelet function achieves the highest accuracy at different decomposition scales. For ZW225 dataset, db8 wavelet is chosen, when the decomposition scale are 3, 4 and 5, the overall prediction accuracy of subcellular localization are 100%, 100% and 99.56%, respectively. When the decomposition scale is 4, the highest prediction accuracy of 100% is achieved by choosing db8 wavelet, which is 2.22% higher than the prediction accuracy of bior2.4 wavelet. In this paper, the CL317 and ZW225 protein datasets are selected to provide the best performance for the predicted model of subcellular localization of apoptosis proteins. We need to consider the two datasets at the same time under different decomposition scales and different wavelet functions. Good effect of wavelet function and decomposition scale. We need to consider the wavelet function and decomposition scale which obtained the best effect of the two datasets at the same time. In order to more intuitively analyze the best wavelet function and decomposition scale in two datasets, we draw the histogram of the overall prediction accuracy of subcellular localization of apoptosis proteins at different decomposition scales, as shown in Figures 3 and 4.

It can be seen from Figures 3 and 4 that when the db8 wavelet is selected and decomposition scale is 4, the highest overall prediction accuracy of the model can be obtained for datasets CL317 and ZW225. As we all know, wavelet function has many excellent properties, such as compactly support, orthogonality, symmetry, smoothness and high order of vanishing moments. In practice, wavelet function selection has some conflicting constraints, and none of these wavelet functions share simultaneously all of these properties.

As shown in Table 5, when we choose the db8 wavelet function and the decomposition scale is 4, the overall accuracy of the subcellular localization prediction of the two datasets CL317 and ZW225 are the highest, which are 99.37% and 100%, respectively. The db8 wavelet function has good locality characteristics in both time and frequency domain, which can also effectively remove high frequency noise and reduce dimensionality, eliminate the redundant information of protein sequences, reduce the leakage and aliasing of feature information, so that the extracted feature vector can better express the original sequence information, and improve the prediction performance of protein subcellular localization [42].

### Effect of feature extraction algorithm on results

Using the feature extraction algorithm to extract the effective information of protein sequences is an important step in protein subcellular localization prediction. PseAAC algorithm is a feature extraction algorithm based on amino acid residues. PsePSSM is a feature extraction algorithm based on sequence homology. By combining the PseAAC algorithm with the PsePSSM algorithm, more feature information of the protein sequence will be obtained, but it will also bring more redundant information. The use of 2-D wavelet denoising can effectively remove the redundant information in the protein sequence and extract the characteristic signals of each protein itself. In this paper, we compare the different effect of feature extraction methods on prediction results, and select the optimal feature extraction algorithm.

Among them, when PseAAC is used for feature extraction, *λ =* 15 is selected. When PsePSSM is used for feature extraction, *ξ* = 10 is selected. When the two algorithms PseAAC and PsePSSM are combined, the optimal *λ =* 15 and *ξ =* 10 are chosen to generate 255 dimension feature vector. In the 2-D wavelet denoising, we use the db8 wavelet function and the decomposition scale is 4. Four kinds of feature extraction methods using the linear kernel function with SVM to classify and obtain the different prediction results of the two apoptosis protein datasets CL317 and ZW225, as shown in Tables 6 and 7.

**Table 6:** Prediction results of subcellular localization by four different feature extraction methods on dataset CL317

Algorithm	Locations						
	Jackknife test (%)						
	Cy	Me	Mi	Se	Nu	En	OA
PseAAC	88.39	90.91	73.53	41.18	78.85	95.74	84.23
PsePSSM	91.96	89.09	88.24	82.35	88.46	97.87	90.85
PseAAC-PsePSSM	90.18	89.09	85.29	88.24	90.38	93.62	89.91
PseAAC-PsePSSM-WD	100	100	94.12	100	100	100	99.37

**Table 7:** Prediction results of subcellular localization by four different feature extraction methods on dataset ZW225

Algorithm	Locations				
	Jackknife test (%)				
	Cy	Me	Mi	Nu	OA
PseAAC	85.71	87.64	64.00	63.41	80.00
PsePSSM	84.29	91.01	72.00	85.37	85.78
PseAAC-PsePSSM	87.14	93.26	80.00	80.49	87.56
PseAAC-PsePSSM-WD	100	100	100	100	100

**Figure 5:**
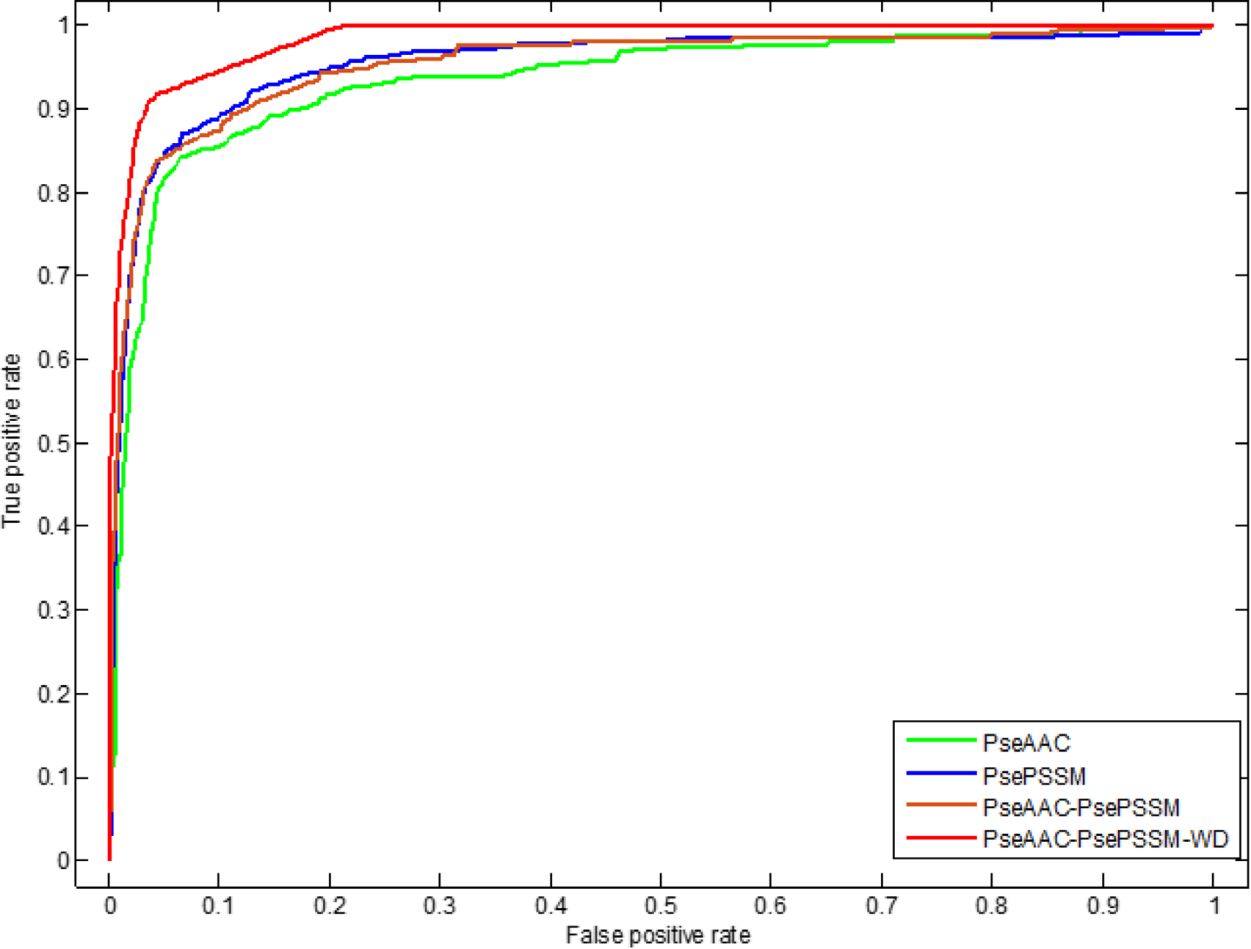
This graph shows the ROC curves of CL317 dataset

**Figure 6:**
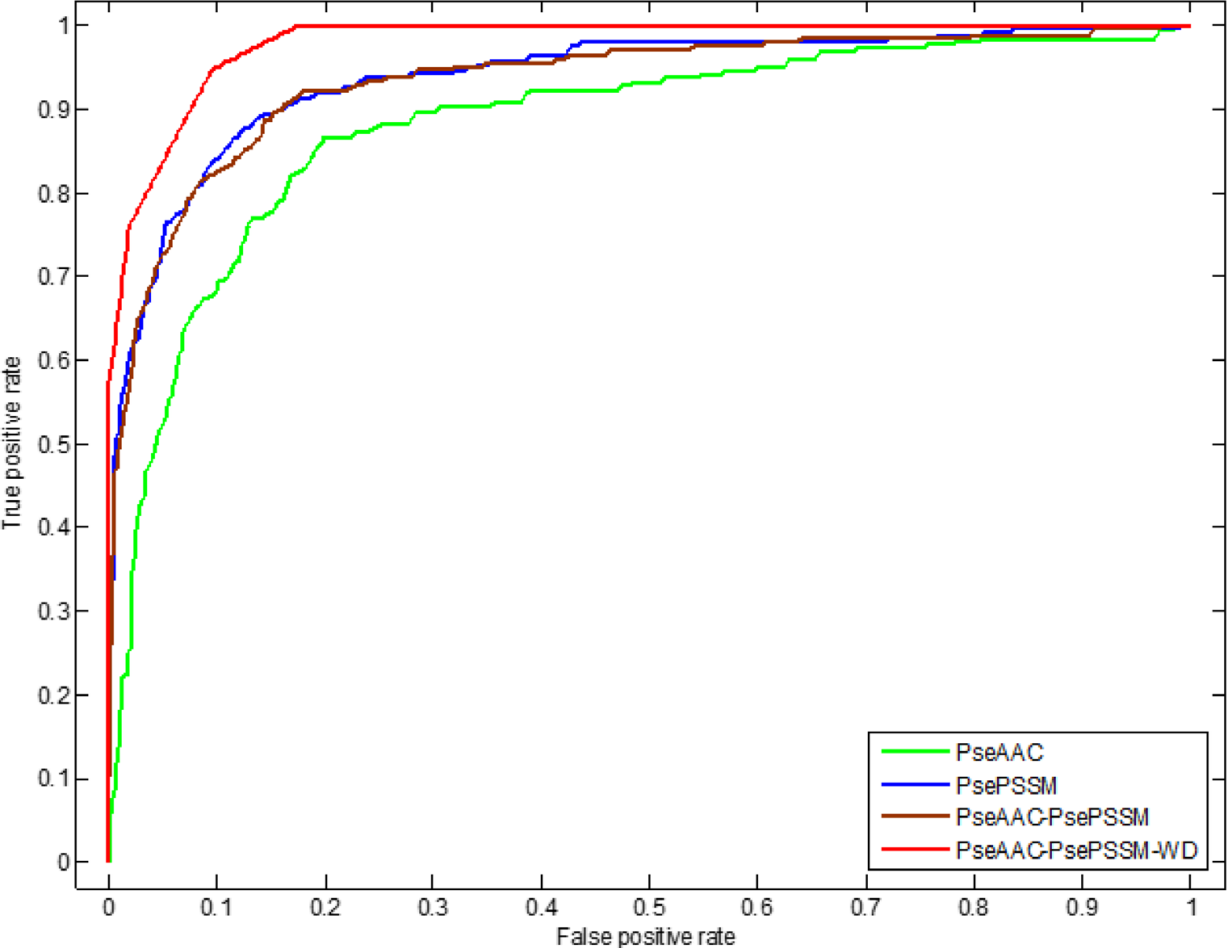
This graph shows the ROC curves of ZW225 dataset

In addition, we use ROC (receiver operating characteristic) curve to compare the robustness of the model under different feature extraction algorithms. In general, ROC curve is applicable to evaluate the prediction performance of a binary classifier, but apoptosis proteins subcellular localization prediction is a multi-class prediction problem. We first use the one-versus-rest (OVR) strategy to transform the multi-classification problem into two-classification problems. And then for these two-classification true positive rate and false positive rate, the average of them are taken as the final result. Figures 5 and 6 are the ROC curves obtain by four different feature extraction methods for the CL317 dataset and ZW225 dataset, respectively.

As can be seen from Table 6, different feature extraction algorithms make the prediction accuracy of each protein in CL317 data set different. The overall prediction accuracy is 84.23% by using PseAAC algorithm, which is 6.62% lower than that of using PsePSSM algorithm. The prediction accuracy of the PseAAC-PsePSSM algorithm is 89.91%, which is 0.94% lower than that of PsePSSM algorithm. The possible reason is that the two algorithms combine to bring more redundant information for protein sequence, which leads to the decrease of prediction accuracy. The prediction accuracy of the six kinds of proteins in CL317 dataset has been improved obviously by 2-D wavelet denoising on feature data after combining two algorithms, and the overall prediction accuracy of the model has reach 99.37%. For the secreted protein in the CL317 dataset, the prediction accuracy of the PseAAC algorithm is 41.18%, and the accuracy is significantly improved using the other three feature extraction algorithms. The prediction accuracy of the PseAAC-PsePSSM-WD algorithm is 100% for the secreted protein, which is 58.82% higher than PseAAC algorithm. It can be seen from Figure 5 that for the CL317 dataset, the coverage area of the ROC curve using the PseAAC-PsePSSM-WD feature extraction method is the largest, and the AUC value is 0.9842, which is significantly higher than that of the other three methods. The AUC values of PseAAC, PsePSSM and PseAAC-PsePSSM methods are 0.9358, 0.9540 and 0.9532, respectively.

As can be seen from Table 7, different feature extraction algorithms make the predictive accuracy of each type of protein in the ZW225 dataset different. Using the PseAAC algorithm, the overall prediction accuracy is the lowest, the prediction accuracy is 80.00%, which is 5.78% lower than that of using the PsePSSM algorithm. After the PseAAC algorithm and PsePSSM algorithm are fused, the overall prediction accuracy rate is 87.56%, which is higher than the overall prediction accuracy rate when the two algorithms are used respectively. We use the 2-D wavelet denoising method to combine the two algorithms to further extract the sequence data, and the prediction accuracy of the four kinds of proteins in the ZW225 dataset has been improved remarkably, and the overall prediction accuracy of the model has reached 100%. It can be seen from Figure 6 that for the ZW225 dataset, the AUC value of the ROC curve is 0.9805 using the PseAAC-PsePSSM-WD feature extraction method, and the robustness of the algorithm is the best, obviously higher than the other three methods. The AUC values of the PseAAC, PsePSSM and PseAAC-PsePSSM methods are 0.8823, 0.9391 and 0.9342, respectively. By comparing the influence of different feature extraction algorithms on the prediction results and comparing the robustness of four different feature extraction algorithms from the ROC curve, we determined that PseAAC-PsePSSM-WD is the best feature extraction algorithm for this study.

### Effect of kernel function on results

**Figure 7:**
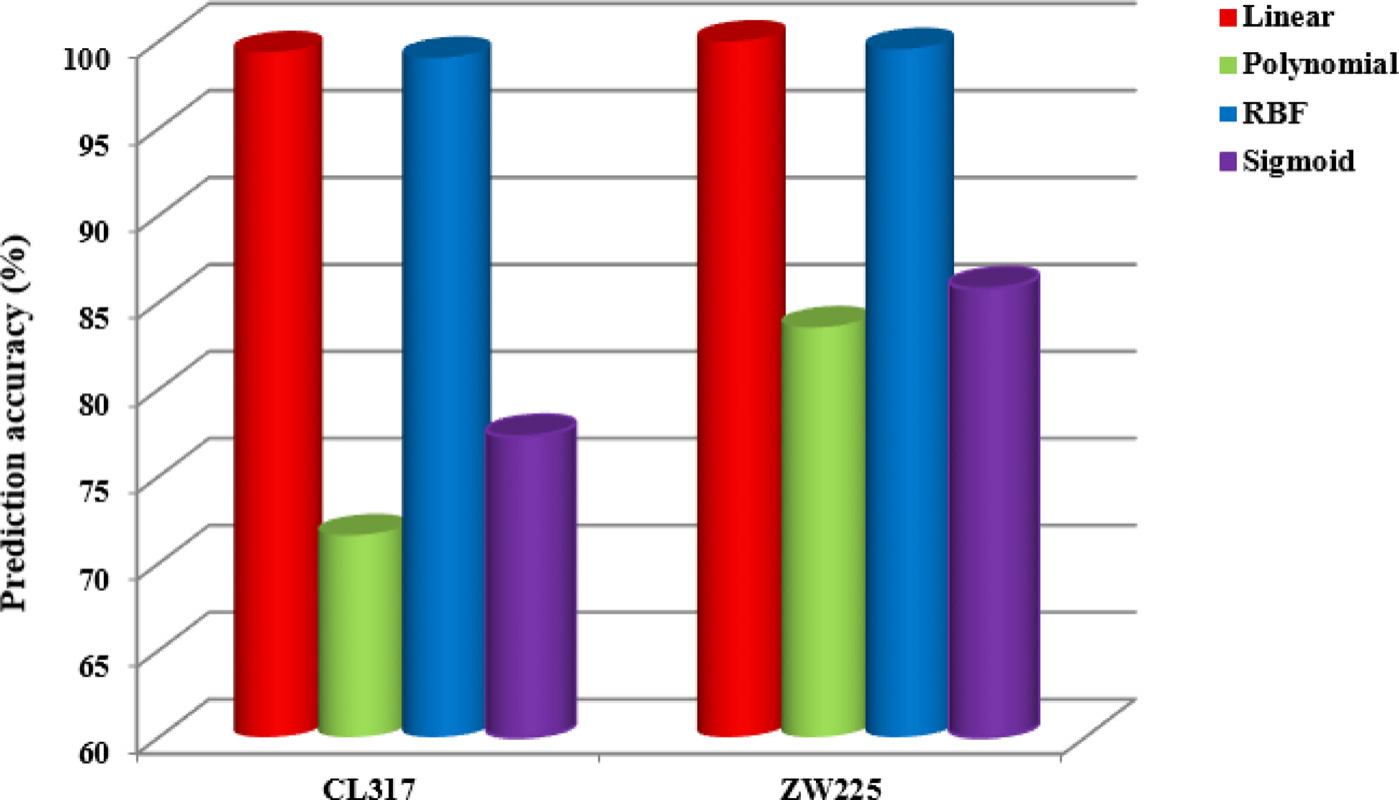
The overall prediction accuracy of two apoptosis datasets CL317 and ZW225 under four different kernel functions

When SVM is used to classify, the selection of kernel function will have important influence on the prediction results. In this paper, using PseAAC and PsePSSM algorithms for feature extraction of protein sequence. When PseAAC is used for feature extraction, *λ =* 15 is chosen. When PsePSSM is used for feature extraction, *ξ =* 10 is chosen and 2-D wavelet denoising using db8 wavelet function, the decomposition scale of 4, select the different kernel functions to get prediction results of subcellular localization of two apoptosis datasets, as is shown in Tables 8 and 9. Where, 0 denotes a linear kernel function, 1 denotes a cubic polynomial kernel function, 2 denotes an RBF kernel function, and 3 denotes a sigmoid kernel function. Figure 7 shows the variation of the overall prediction accuracy of the two datasets under different kernel functions.

**Table 8:** Prediction results of subcellular localization of the CL317 dataset under different kernel functions

Locations	Functions			
	Jackknife test (%)			
	0	1	2	3
Cy	100	100	100	87.50
Me	100	30.91	100	80.00
Mi	94.12	0	91.18	61.76
Se	100	94.12	100	0.00
Nu	100	71.15	100	73.08
En	100	95.74	100	93.62
OA	99.37	71.61	99.05	77.29

**Table 9:** Prediction results of subcellular localization of the ZW225 dataset under different kernel functions

Locations	Functions			
	Jackknife test (%)			
	0	1	2	3
Cy	100	84.29	100	100
Me	100	100	98.88	86.52
Mi	100	0.00	100	52.00
Nu	100	97.56	100	80.49
OA	100	83.56	99.56	85.78

It can be seen from Figure 7 that the prediction effect by linear kernel function and RBF kernel function is better than that of the other two kernel functions when using SVM for datasets CL317 and ZW225. As can be seen from Table 8, the accuracy of the cytoplasmic proteins, membrane proteins, secreted proteins, nuclear proteins and endoplasmic reticulum proteins in the dataset CL317 are 100% using the linear kernel function, and the overall prediction accuracy of the model is 99.37%. The overall prediction accuracy is 99.05% using the RBF kernel function, which is 0.32% lower than that using linear kernel function. In addition, the overall prediction accuracy of polynomial kernel function and sigmoid kernel function are 71.61% and 77.29%, respectively, which are 27.76% and 22.08% lower than that of linear kernel function respectively. It can be seen from Table 9 that the overall prediction accuracy is 100% in the dataset ZW225 using the linear kernel function, which are 16.44%, 0.44% and 14.22% higher than using polynomial kernel function, RBF kernel function and sigmoid kernel function respectively. Considering the influence of different kernel functions on the datasets CL317 and ZW225, the linear kernel function is used as the kernel function of SVM algorithm.

### Selecting classification algorithms

Because of the large number of protein sequences and hidden information which is difficult to reveal, the performance of the prediction algorithm has a high demand. In recent years, pattern recognition methods such as statistics and machine learning have been widely used in prediction algorithms. In this study, we mainly studied five classification algorithms: support vector machine (SVM), k-nearest neighbor (KNN), random forest (RF), naïve Bayes, decision tree (DT). SVM algorithm uses linear kernel function, KNN algorithm using Euclidean distance, the number of neighbors is 3, the number of decision trees selected in RF is 100, naïve Bayes algorithm and DT algorithm adopt default parameters.

For the CL317 and ZW225 apoptosis protein datasets, the PseAAC and PsePSSM algorithms are used to extract features of the protein sequences in the datasets. The 2-D wavelet denoising method are used to further process for the feature data, the db8 wavelet function is still used, and the decomposition scale is 4. Under the jackknife test, the main predictions of the five different classification algorithms are shown in Tables 10 and 11. Five classification algorithms for the two datasets CL317 and ZW225 subcellular localization prediction of specific results see the Supplementary Tables 3 and 4.

**Table 10:** Prediction results of subcellular localization of the CL317 dataset under different classification algorithms

Classifiers	Evaluate			
	Jackknife test			
	Sens (%)	Spec (%)	MCC	OA (%)
SVM	99.02	99.87	0.9908	99.37
KNN	99.01	99.79	0.9889	99.05
RF	97.26	99.52	0.9690	97.79
Naïve Bayes	89.61	97.51	0.8629	88.33
DT	93.61	98.98	0.9164	94.64

**Table 11:** Prediction results of subcellular localization of the ZW225 dataset under different classification algorithms

Classifiers	Evaluate			
	Jackknife test			
	Sens (%)	Spec (%)	MCC	OA (%)
SVM	100	100	1	100
KNN	93.72	98.77	0.9447	96.89
RF	98.72	99.65	0.9871	99.11
Naïve Bayes	95.35	98.04	0.9087	93.78
DT	97.44	99.34	0.9710	98.22

It can be seen from Table 10 that for the dataset CL317, SVM algorithm is used as the prediction algorithm of the model, the overall prediction accuracy of the model is 99.37%, and the sensitivity, specificity and Matthew's correlation coefficient (MCC) are 99.02%, 99.87% and 0.9908, respectively. The results of these evaluation indicators are superior to the other four classification algorithms. Using the naïve Bayes algorithm, the overall prediction accuracy of the model is 88.33%, which is 11.04% lower than that using the SVM algorithm.

**Figure 8:**
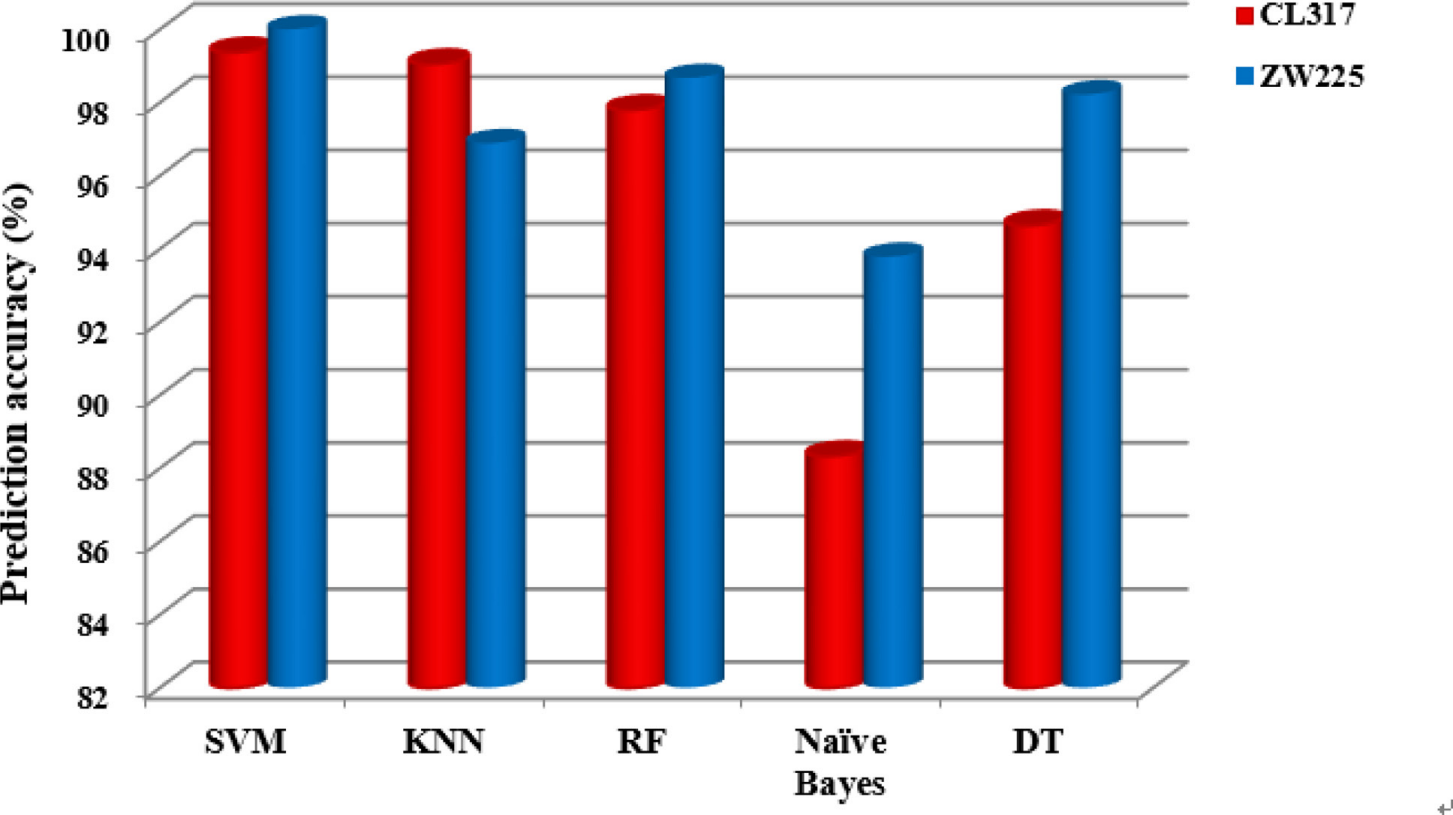
The overall prediction accuracy of subcellular localization of the five classification algorithms for datasets CL317 and ZW225

It can be seen from Table 11 that for the dataset ZW225, the SVM algorithm is used as the prediction algorithm of the model, and the overall prediction accuracy of the model is 100%, and the sensitivity, specificity and Matthew's correlation coefficient (MCC) are 100%, 100% and 1, respectively. The results of these evaluation indicators are superior to the other four classification algorithms. Based on the influence of different prediction algorithms on the prediction results, Figure 8 shows the change of the overall prediction accuracy in datasets CL317 and ZW225 under different classification algorithms.

It can be clearly seen from Figure 8 that the overall prediction accuracy is significantly higher than that of the other four prediction algorithms when SVM is used as the prediction algorithm in the two datasets. Because of the fast computation speed of SVM, the extraction ability of the training set implicit information is strong, and it has the advantages of excellent generalization performance, which can effectively avoid over-fitting. Considering that we selected the SVM classification algorithm combined with the PseAAC-PsePSSM-WD model constructed by 2-D wavelet denoising to predict the apoptosis proteins subcellular localization.

### Performance of prediction model

**Figure 9:**
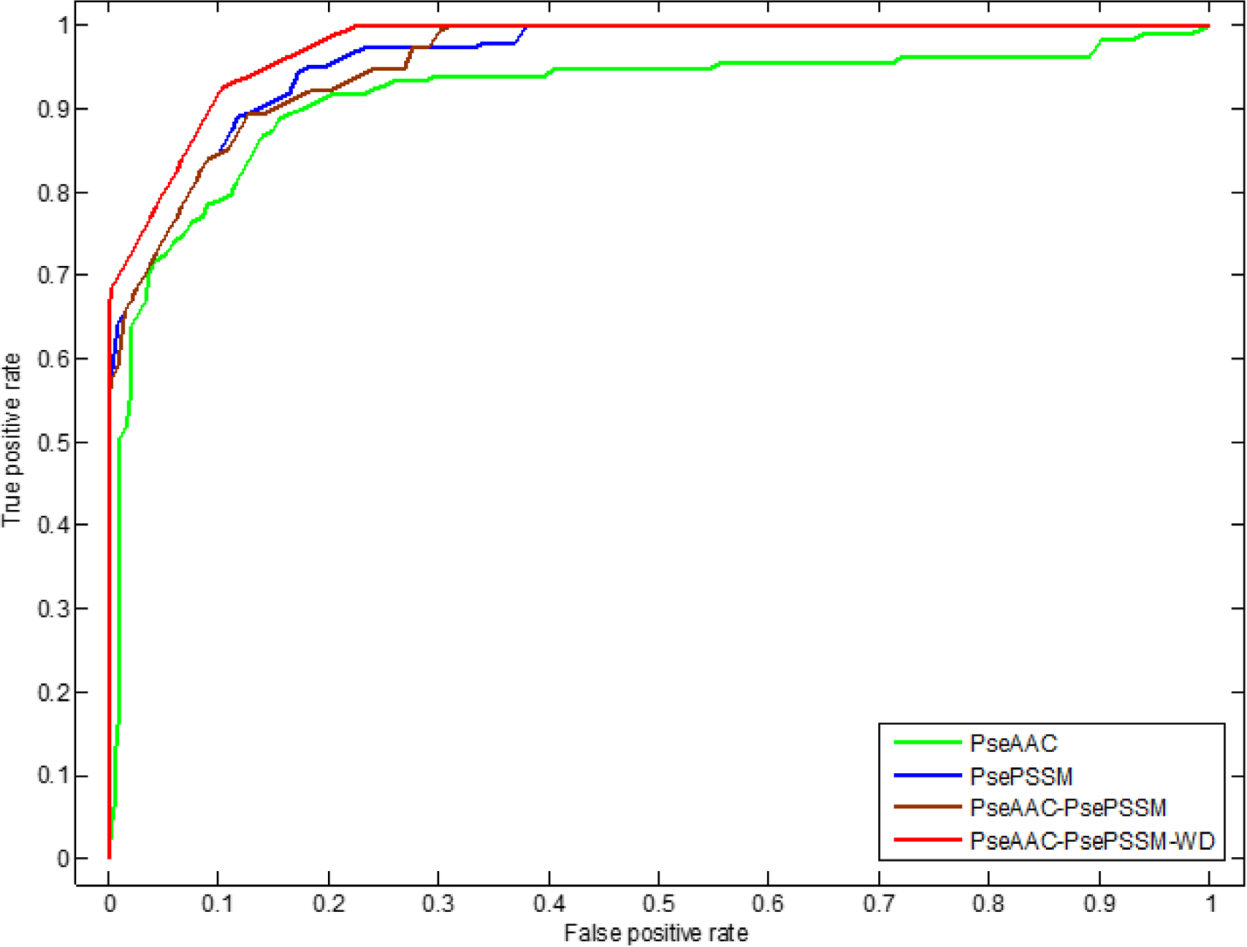
This graph shows the ROC curves of ZD98 dataset

In this paper, the protein sequence features are extracted by fusing PseAAC and PsePSSM, and then the subcellular localization of apoptosis proteins is predicted by SVM based on 2-D wavelet denoising. According to the above analysis, PseAAC is used to extract feature information, *λ =* 15 is chosen, when PsePSSM is used to extract feature information, *ξ* = 10 is chosen. In the process of 2-D wavelet denoising, the default threshold is obtained by using the wavelet function, the wavelet function is db8, the decomposition scale is 4, and the linear kernel function is selected as the kernel function of SVM. In this paper, self-consistency and jackknife test are used to verify the datasets CL317 and ZW225. The main results are shown in Tables 12 and 13. In order to further validate the actual predictive ability of the model, we use the independent testing dataset ZD98 to evaluate our proposed prediction model, the results are shown in Table 14. In addition, since the ROC curve can test the robustness of the model, Figure 9 is the ROC curve obtained by four different feature extraction methods for the ZD98 dataset.

**Table 12:** Prediction performance of different test method for protein subcellular localization on the CL317 dataset

Locations	Test					
	Self-consistency test			Jackknife test		
	*Sens* (%)	*Spec* (%)	MCC	*Sens* (%)	*Spec* (%)	MCC
Cy	100	100	1	100	100	1
Me	100	100	1	100	99.62	0.9891
Mi	100	100	1	94.12	100	0.9667
Se	100	100	1	100	100	1
Nu	100	100	1	100	99.62	0.9886
En	100	100	1	100	100	1
OA	100			99.37		

**Table 13:** Prediction performance of different test method for protein subcellular localization on the ZW225 dataset

Locations	Test					
	Self-consistency test			Jackknife test		
	*Sens* (%)	*Spec* (%)	MCC	*Sens* (%)	*Spec* (%)	MCC
Cy	100	100	1	100	100	1
Me	100	100	1	100	100	1
Mi	100	100	1	100	100	1
Nu	100	100	1	100	100	1
OA	100			100		

**Table 14:** Prediction performance of different test method for protein subcellular localization on the ZD98 dataset

Locations	Test					
	Self-consistency test			Jackknife test		
	*Sens* (%)	*Spec* (%)	MCC	*Sens* (%)	*Spec* (%)	MCC
Cy	100	100	1	100	100	1
Me	100	100	1	100	100	1
Mi	100	100	1	100	98.82	0.9579
Other	100	100	1	91.67	100	0.9519
OA	100			98.98		

It can be seen from Tables 12 and 13 that the datasets CL317 and ZW225 are tested by the self-consistency test method respectively. The overall prediction accuracy is 100%, and the sensitivity, specificity and Matthew's correlation coefficient of each protein in the two datasets all reach 100%, 100% and 1, respectively. The experimental results show that the proposed model can grasp the autocorrelation of subcellular localization of each type of apoptosis proteins. Jackknife method are used to test two datasets. For the dataset CL317, the sensitivity of cytoplasmic proteins, membrane proteins, secreted proteins, nuclear proteins and endoplasmic reticulum proteins all reached 100%. The sensitivity of mitochondrial protein is 94.12%. The specificity of cytoplasmic proteins, secreted proteins and endoplasmic reticulum proteins reached 100%, and MCC is 1. The overall prediction accuracy of PseAAC-PsePSSM-WD model is 99.37%. For the dataset ZW225, the overall prediction accuracy of the PseAAC-PsePSSM-WD model is 100%. The sensitivity, specificity, and Matthew's correlation coefficients of each protein are 100%, 100%, and 1, respectively, and the best results are obtained.

It can be seen from Table 14 that the self-consistency method is used to test dataset ZD98, the overall prediction accuracy is 100%. The sensitivity, specificity and Matthew's correlation coefficient of each class proteins in the dataset are 100%, 100% and 1, respectively. The experimental results show that the proposed model can grasp the autocorrelation of subcellular localization of each class of apoptosis proteins. The most rigorous jackknife method is used to test the dataset. The sensitivity of cytoplasmic proteins, mitochondrial proteins and plasma membrane proteins all reached 100%, and the specificity of cytoplasmic proteins, other proteins and plasma membrane proteins all reached 100%. The overall prediction accuracy of PseAAC-PsePSSM-WD model is 98.98%. It can be seen from Figure 9 that for the ZD98 dataset, the coverage area of the ROC curve using the PseAAC-PsePSSM-WD feature extraction method is the largest, and the AUC value is 0.9760, which is significantly higher than that of the other three methods. The AUC values of PseAAC, PsePSSM and PseAAC-PsePSSM methods are 0.9152, 0.9615 and 0.9595, respectively. The above results show that the PseAAC-PsePSSM-WD model proposed in this paper is an effective tool for subcellular localization prediction.

### Comparison with other methods

A number of prediction methods have been proposed for the prediction of subcellular localization of apoptosis proteins. In order to evaluate the validity of the PseAAC-PsePSSM-WD model proposed in this paper, based on the jackknife test, we compared the prediction accuracy of the model with the predicton method of the same dataset. Tables 15 and 16 details the comparison of the proposed method and other prediction methods on the datasets CL317 and ZW225, respectively.

**Table 15:** Prediction results with different methods on the CL317 dataset using jackknife test

Methods	Jackknife test (%)						
	Sensitivity for each class						
	Cy	Me	Mi	Se	Nu	En	OA
ID [44]	81.3	81.8	85.3	88.2	82.7	83.0	82.7
ID_SVM [34]	91.1	89.1	79.4	58.8	73.1	87.2	84.2
DF_SVM [45]	92.9	85.5	76.5	76.5	93.6	86.5	88.0
FKNN [35]	93.8	92.7	82.4	76.5	90.4	93.6	90.9
PseAAC_SVM [46]	93.8	90.9	85.3	76.5	90.4	95.7	91.1
EN_FKNN [47]	98.2	83.6	79.4	82.4	90.4	97.9	91.5
DWT_SVM [36]	100	98.2	82.4	94.1	100	100	97.5
APSLAP [48]	99.1	89.1	85.3	88.2	84.3	95.8	92.4
Liu *et al*. [38]	98.2	96.4	94.1	82.4	96.2	95.7	95.9
Auto_Cova [37]	86.4	90.7	93.8	85.7	92.1	93.8	90.0
DCCA coefficient [39]	91.1	92.7	82.4	76.5	80.8	93.6	88.3
PseAAC-PsePSSM-WD	**100**	**100**	94.1	100	100	**100**	**99.4**

**Table 16:** Prediction results with different methods on the ZW225 dataset using jackknife test

Methods	Jackknife test (%)				
	Sensitivity for each class				
	Cy	Me	Mi	Nu	OA
ID_SVM [34]	92.9	91.0	68.0	73.2	85.8
DF_SVM [45]	87.1	92.1	64.0	73.2	84.0
FKNN [35]	84.3	93.3	72.0	85.5	85.8
EN_FKNN [47]	94.3	94.4	60.0	80.5	88.0
DWT_SVM [36]	87.1	93.2	64.0	90.2	87.6
Liu *et al*. [38]	97.1	98.9	96.0	97.6	97.8
Auto_Cova [37]	81.3	93.3	85.7	84.6	87.1
EBGW_SVM [33]	90.0	93.3	60.0	63.4	83.1
Dual-layer SVM [49]	91.4	94.4	76.0	78.1	88.4
PseAAC-PsePSSM-WD	100	100	100	100	**100**

As can be seen from Table 15, for the CL317 dataset, the overall prediction accuracy of this method is 99.4%, 1.9%–16.7% higher than other prediction methods. The overall prediction accuracy of the PseAAC-PsePSSM-WD model is 1.9% higher than that of the DWT_SVM prediction method proposed by Qiu *et al*. [36], which is 16.7% higher than that proposed by Chen *et al*. [44]. The overall prediction accuracy of our model is obviously improved. Besides, the prediction accuracy of cytoplasmic, membrane, secreted, nuclear and endoplasmic reticulum proteins all reached 100% using this method, which is significantly higher than the predictive accuracy of other methods on these protein subcellular. For the secreted proteins, the accuracy of the PseAAC-PsePSSM-WD method is 100% and 41.2% higher than the ID_SVM [34] algorithm. For the mitochondrial proteins, the accuracy of the PseAAC-PsePSSM-WD method is 94.1% and 14.7% higher than the EN_FKNN [47] algorithm. In summary, our proposed method has achieved satisfactory prediction results on CL317 dataset.

As can be seen from Table 16, for dataset ZW225, the overall prediction accuracy of this method is 100%, which is 2.2%–16.9% higher than other prediction methods, 12.4% higher than DWT_SVM prediction method proposed by Qiu *et al*. [36], and 16.9% higher than that proposed by EBGW_SVM [33]. In addition, the prediction accuracy of cytoplasmic proteins, membrane proteins, mitochondrial proteins and nuclear proteins all reached 100% by this method, which is significantly higher than that of other methods on these proteins. For mitochondrial proteins, the prediction accuracy of PseAAC-PsePSSM-WD method is 100%, which is 40.0% higher than that of EBGW_SVM [33], 36.0% higher than that of DF_SVM [45]. It is shown that the model proposed in this paper has excellent robustness for the prediction of mitochondrial proteins in apoptosis proteins. In summary, our proposed method has achieved satisfactory predictions on the ZW225 dataset.

In order to further validate the actual predictive ability of PseAAC-PsePSSM-WD model, we used the independent testing dataset ZD98 and compared with other reported prediction methods. Table 17 shows the prediction results of the subcellular localization on the ZD98 dataset.

**Table 17:** Performance comparison of the independent testing dataset by the jackknife test on the ZD98 dataset

Methods	Jackknife test (%)				
	Sensitivity for each class				
	Cy	Me	Mi	Other	OA
ID [44]	90.7	90.0	92.3	91.7	90.8
ID_SVM [34]	95.3	93.3	84.6	58.3	88.8
DF_SVM [45]	97.7	96.7	92.3	75.0	93.9
FKNN [35]	95.3	96.7	100	91.7	95.9
PseAAC_SVM [46]	95.3	93.3	92.3	83.3	92.9
DWT_SVM [36]	95.4	93.3	53.9	91.7	88.8
APSLAP [48]	95.3	90.0	100	91.7	94.9
Liu et al. [38]	95.3	100	100	91.7	96.9
EBGW_SVM [33]	97.7	90.0	92.3	83.3	92.9
DCCA coefficient [39]	93.0	86.7	92.3	75.0	88.9
PseAAC-PsePSSM-WD	100	100	100	91.7	**99.0**

As can be seen from Table 17, the overall prediction accuracy of PseAAC-PsePSSM is 99.0%, which is 2.1%–10.2% higher than other prediction algorithms, 10.2% higher than DWT_SVM method proposed by Qiu *et al*. [36] and 2.1% higher than SVM_RFE method proposed by Liu *et al*. [38]. In addition, the prediction accuracy of cytoplasmic proteins, membrane proteins and mitochondrial proteins is 100% using our method, which is significantly higher than that of other methods on these proteins. For the mitochondrial proteins, the prediction accuracy of DWT_SVM [36] is 53.9%, 46.1% lower than the PseAAC-PsePSSM-WD method. It indicated that our approach for mitochondrial proteins prediction has achieved good results. In addition, the method proposed in this paper on the other proteins accuracy rate is 91.7%, due to ZD98 dataset, the other proteins has 12 sequences, and only one protein sequence belonging to the other class is predicted to be wrong, but it also achieves the highest prediction accuracy. It indicates that the model of this paper has excellent properties for the prediction subcellular localization of apoptosis proteins. In conclusion, our proposed method has achieved satisfactory prediction results.

## CONCLUSIONS

With the advent of the big data age, massive protein sequences are exponentially growing into the database. The traditional method of biological experiments has been unable to meet the needs of life science research, so it is more and more important to study protein subcellular localization prediction method based on machine learning. In this paper, a novel protein subcellular localization prediction method is proposed. Three apoptosis protein datasets are selected, fuse Chou's PseAAC and PsePSSM algorithms to carry out feature extraction on protein sequences, and subcellular localization of proteins is predicted by SVM algorithm based on 2-D wavelet denoising. PseAAC feature extraction can avoid the loss of protein sequence-order information, and obtains more detailed protein sequence information. PseAAC feature extraction not only contains the frequency of various amino acids in the protein sequences, but also makes full use of the order of amino acids in the sequence and various physical and chemical properties. PsePSSM feature extraction can obtain the evolutionary information of the protein sequences and also retain the sequence information of the amino acid residues in the protein sequences. Wavelet transform has the characteristics of multiresolution analysis, and can have the ability to characterize the local characteristics of signal in the time domain and frequency domain. Therefore, 2-D wavelet denoising can effectively remove redundant information in the protein sequences, and extract the characteristic signals of each protein itself, which is very important to ensure the high prediction accuracy. SVM classification algorithm can deal with high-dimensional data, to avoid over-fitting and effective removal of non-support vector. By the most rigorous jackknife test, the overall prediction accuracies of the three benchmark datasets reach 99.37%, 100% and 98.98%, respectively, and compared with other existing methods. The experimental results show that our proposed method not only can effectively improve the prediction accuracy of protein subcellular localization, but also is expected to be used for the prediction of other attributes of proteins. We expect that the proposed method can be a powerful tool in the field of bioinformatics, proteomics and molecular biology. Since user-friendly and publicly accessible web-servers represent the future direction for developing practically more useful predictors [42, 50], we shall make efforts in our future work to provide a web-server for the method presented in this study.

## MATERIALS AND METHODS

### Datasets

In order to facilitate the comparison with the previous works, three widely used benchmark datasets: CL317, ZW225 and ZD98 are adopted in our work. The CL317 dataset consists of 317 apoptosis proteins constructed by Chen and Li [34] with six subcellular locations, including 112 cytoplasmic proteins, 55 membrane proteins, 34 mitochondrial proteins, 17 secreted proteins, 52 nuclear proteins and 47 endoplasmic reticulum proteins. The ZW225 dataset consists of 225 apoptosis proteins constructed by Zhang *et al*. [33] with 41 nuclear proteins, 70 cytoplasmic proteins, 25 mitochondrial proteins and 89 membrane proteins. The ZD98 dataset consists of 98 apoptosis proteins constructed by Zhou and Doctor [31] with 43 cytoplasmic proteins, 30 plasma membrane-bound proteins, 13 mitochondrial proteins and 12 other proteins. The protein sequences in the three datasets are extracted from SWISS-PROT database (version 49.5), and the accession numbers can be found in the literatures [31, 33, 34]. In this study, datasets CL317 and ZW225 are selected as the training datasets, used to select the parameters of the prediction model, and the ZD98 dataset is selected as an independent testing dataset, used to test the applicability of the prediction model.

### Feature extraction of protein sequences

### PseAAC

In order to avoid losing sequence-order information of proteins, Chou [51] proposed pseudo-amino acid composition (PseAAC) approach to solve the problem of extraction of feature vectors in protein sequences. They developed an online server [52] that calculates the composition of pseudo-amino acids at http://www.csbio.sjtu.edu.cn/bioinf/PseAA/. On this server, the researchers can obtain a variety of different forms of PseAAC by selecting different parameters. At present, the researchers have made extensive use of the PseAAC methods in predicting protein structure [50, 53–57], protein function prediction [58, 59], protein-protein interaction prediction [60–62], protein subcellular localization prediction [42, 63–66] and protein posttranslational modification sites [67] and so on.

According to Chou's PseAAC discrete model, the protein sequence could be represented by (20 + *λ – D*) (dimensional) vector.

$$P={\left[p_{1},p_{2},...,p_{20},p_{20+1},...,p_{20+\lambda }\right]}^{T}$$ (1)

where the (20 + *λ*) components are given by

$$p_{u}=\left\{ \begin{array}{l} \frac{f_{u}}{\sum_{u=1}^{20}f_{u}+\omega \sum_{k=1}^{\lambda }\tau_{k}\;},\;1\leq \text{u}\leq 20\\ \frac{\omega \tau_{u-20}}{\sum_{u=1}^{20}f_{u}+\omega \sum_{k=1}^{\lambda }\tau_{k}},\;20\;+\;1\;\leq \text{ u }\leq \;20\;+\;\lambda \end{array}\right.$$ (2)

where *ω* is the weight factor, which was set at 0.05 in Ref. [51]. *τ_k_* is the *k*-tier sequence correlation factor, which reflects the sequence-order information. *f_u_* is the occurrence frequency of *u*(*u* = 1, 2, …, 20) amino acid in proteins. As can be seen from the above formula, the first 20 dimension of the feature vector *P* is the amino acid composition and the posterior *λ* dimension is the sequence correlation factor reflecting the different levels of amino acid sequence information. Sequence correlation factor can be calculated from the hydrophobicity value, hydrophilicity value, and side chain mass of the amino acids residue. Because the length of the shortest protein sequence is 50 amino acids in the three benchmark datasets, the maximum value allowed for *λ* in Eqs. (1) and (2) is 49.

In this study, we used PseAAC online server developed by Shen and Chou [52] to carry out feature extraction of protein sequences. On this server, by selecting different parameters, the optimal λ can be determined from the accuracy of the prediction results.

### PsePSSM

Jones *et al*. [68] proposed a position specific scoring matrix (PSSM), it uses an iterative BLAST search method to discover more protein sequences that have an evolutionary relationship with the search sequence. This paper mainly used the localization of BLAST to obtain the PSSM of the protein sequence. The BLAST package was downloaded from ftp://ftp.ncbi.nlm.nih.gov/blast/release/LATEST/ to the local, you can call the corresponding executable file by the form of the command line. This paper downloaded a non-redundant (nr) protein database provided by NCBI (ftp://ftp.ncbi.nih.gov/blast/db/nr), which contains about 85,107,862 protein sequences. The PSI-BLAST [69] program to search the non-redundant (NR) database through three iterations with 0.001 as the E-value cutoff for multiple sequence alignment against the sequence of the protein *P*, which has *L* × 20 scores as shown in Eq.(3).

$$P_{PSSM}=\left(\begin{array}{l} P_{1,1}\;P_{1,2}\;\cdots \;P_{1,20}\\ \;\vdots \;\vdots \;\vdots \;\vdots \\ P_{i,1}\;P_{i,2}\;\cdots \;P_{i,20}\\ \;\vdots \;\vdots \;\vdots \;\vdots \\ P_{L,1,}\;P_{L,2}\;\cdots \;P_{L,20} \end{array}\right)$$ (3)

where *L* is the length of the protein sequence, the *P_i,j_* represents the score of the amino acid residue in the *i* th of the protein sequence being mutated to amino acid type *j* during the evolution process. The rows of matrix represent the positions of the sequence and the columns of the matrix represent the 20 types original amino acids. PSSM scores are generally shown as positive or negative integers.

In this work, the PSSM matrix is normalized, and the elements in the PSSM matrix are transformed between 0 and 1 using the following sigmoid function:

$$f(x)=1/(1+e^{-x})$$ (4)

Because a protein sequence generates a *L* × 20 matrix and the length *L* of the protein sequence in the dataset is different, the PSSM matrix of different protein sequences is transformed into a uniform vector of dimensions by the following formula.

$${\bar{P}}_{PSSM}=(\bar{P_{1}},\bar{P_{2}},\cdots ,\bar{P_{20}})$$ (5)

where $\bar{P_{j}}$ represents the average score of the amino acid residues in the protein *P* being mutated to amino acid type *j* during the evolution process, that is, $\bar{P_{j}}$ represents the composition of the amino acid type *j* in the PSSM. ${\bar{P}}_{PSSM}$ is denoted by PSSM-ACC [70]. However, this method only takes into account the average score of the amino acid residues in the protein sequence changing into the amino type *j*, without taking into account the sequence-order information of the amino acid residues in the protein sequence. Due to this drawback, pseudo-position specific scoring matrix (PsePSSM) was proposed [71], and then obtained the PsePSSM features through the following equations [50]:

$$P_{PsePSSM}^{\xi }={\left(\theta_{1}^{\xi },\theta_{2}^{\xi },\cdots ,\theta_{j}^{\xi },\cdots \theta_{20}^{\xi }\right)}^{T}$$ (6)

$$\theta_{j}^{\xi }=\frac{1}{L-\xi }\sum_{i=1}^{L-\xi }(P_{i,j}-P_{\left(i+\xi ),j\right)}{)}^{2}\;(j=1,2,\cdots 20;\;\xi <L,\;\xi \neq 0)$$ (7)

where $\bar{P_{j}}$ is the correlation factor of amino acid type *j*, whose contiguous distance is *ξ* along the protein sequence. Then, the PsePSSM feature vector can be expressed as follows:

$$P_{PsePSSM}={\left(\bar{P_{1}},\bar{P_{2}},\cdots ,\bar{P_{20}},\theta_{1}^{1},\theta_{2}^{1},\cdots ,\theta_{20}^{1},\cdots ,\theta_{1}^{\xi },\theta_{2}^{\xi },\cdots ,\theta_{20}^{\xi }\right)}^{T}$$ (8)

It can be seen from the formula (8) that a protein sequence PsePSSM generates a 20 + 20 × *ξ* dimension feature vector. This algorithm transforms the protein sequences of different lengths in the dataset into dimension united vector by feature extraction, which is convenient to facilitate the implementation of the next algorithm.

### Two-dimensional wavelet denoising

In recent years, wavelet analysis has attracted wide attention in the field of bioinformatics research [36, 72–74], especially for protein sequence analysis and structural prediction [75–76]. It has the characteristics of multiresolution analysis, and has the ability to characterize the local characteristics of the signal in the time domain and frequency domain. It is a time-frequency localized analysis method in which the window size is fixed but the shape can be changed and the time window and frequency window can be changed.

Wavelet denoising (WD) is one of the important applications of wavelet analysis. For the biological signal, their energy is highly concentrated in a small amplitude of the larger wavelet coefficients or wavelet packet coefficients, due to its randomness of the noise, the energy is more evenly distributed in the wavelet transformation domain. So threshold method is used to suppress the noise to the maximum while preserving the signal in the transform domain [77, 78]. The decomposition of the protein amino acid signals by wavelet analysis can effectively remove the redundant information and extract the characteristic signals of each protein itself, which is very important for ensuring the high prediction accuracy [79].

A two-dimensional (2-D) model with noise [77, 80] can be expressed as:

s(i,j)=f(i,j)+σe(i,j)                                 i,j=1,2,3,⋯m−1 (9)

where (*i*, *j*) is the size of the signal, *f* (*i*, *j*) is the real signals, *e* (*i*, *j*) is the noise, *s* (*i*, *j*) is the signals with noise, and σ is the noise intensity. The purpose of wavelet denoising is to suppress noise *e* (*i*, *j*) and recover real signals *f* (*i*, *j*).

In this paper, we fuse PseAAC and PsePSSM algorithms to extract the features of each protein sequence in the dataset, so that each sequence generates (20 + *λ*) + (20 + 20 × *ξ*) dimension features. For apoptosis protein datasets CL317 and ZW225, the 317 × ((20 + *λ*) + (20 + 20 × *ξ*)) matrix and 225 × ((20 + *λ*) + (20 + 20 × *ξ*)) matrix is generated respectively. The each matrix is treated as a two-dimensional signal, and the entire 2-D signal is denoised.

Two-dimensional wavelet denoising steps are as follows:

(1) Wavelet decomposition of 2-D signal. Using 2-D wavelet transform, make 2-D data decompose to the four scales spatial frequency band, that is, low-frequency scale spatial frequency band signals *f* ^1^, horizontal scale spatial frequency band signals *d^H^*, vertical scale spatial frequency band signals *d^H^*, and the diagonal scale spatial frequency band signals *d^D^*. It is orthogonal decomposition process on different scales, that is $f=f^{1}⊕d^{H}⊕d^{V}⊕d^{D}$ . The low-frequency signals *f^1^* can also continue to be decomposed into four directions: the signals of apoptosis proteins on the low-frequency scale space *f* ^2^, the signals on the horizontal scale spatial band *d*^1,*H*^, the signals on the vertical scale spatial frequency band *d*^1,*V*^, and the signals on the diagonal scale spatial frequency band *d*^1,*D*^. The decomposition process is still orthogonal which is $f^{1}=f^{2}⊕d^{1,H}⊕d^{1,V}⊕d^{1,D}$ . Since the signals energy of the apoptosis protein is limited, so the number of decomposition of the 2-D wavelet is also limited, and the process of the *n*th wavelet decomposition is $f^{n-1}=f^{n}⊕d^{n-1,H}⊕d^{n-1,V}⊕d^{n-1,D}$.

(2) Threshold quantization of the decomposed high-frequency coefficients. Threshold quantization is performed by selecting the appropriate thresholds for the high-frequency coefficients from 1 to the *N* horizontal, vertical and diagonal directions of each layer.

(3) Reconstruct the two-dimensional signals. The 2-D signals are reconstructed according to the low-frequency coefficients of the wavelet decomposition and the high-frequency coefficients after the threshold quantization. The reconstructed signals are the noise reduction signals.

In these three steps, the most critical is how to select the threshold and the threshold quantization. Because the selection of thresholds directly affects the quality of noise reduction, a variety of theoretical and empirical models are proposed, but none of them is general. 2-D signals commonly used threshold models: default threshold determination model, and threshold model determined by Birge-Massart strategy. We can choose the wavelet functions or wavelet packets to confirm the default threshold. In the process of numerical experiment, this study aims at select appropriate methods to improve the overall prediction accuracy.

In the process of noise reduction, the wavelet coefficients were subjected to threshold processing operation. The selection of soft threshold and hard threshold has certain influence on the results of wavelet denoising [81]. Soft threshold is a more smooth way to produce a smoother effect after noise reduction. While hard threshold processing preserves some features such as spikes in the signal, we choose soft threshold wavelet denoising method.

**Figure 10:**
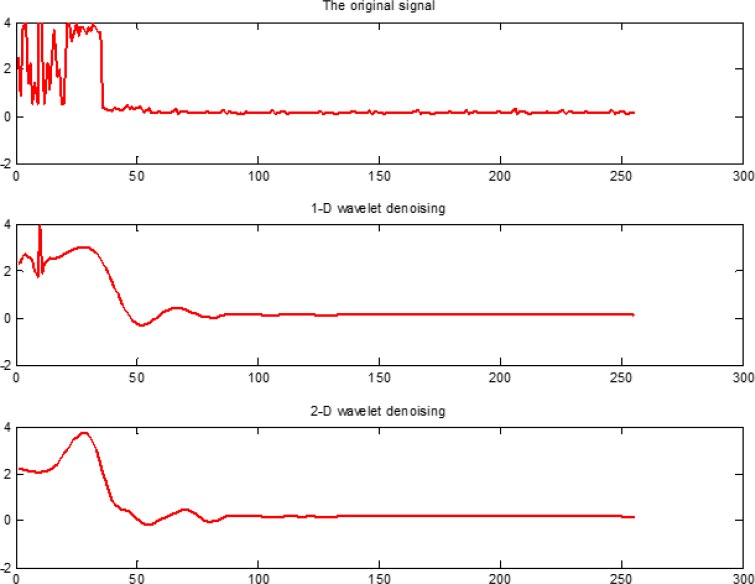
Wavelet denoising using db8 wavelet function and decomposition scale is *j* = 4. The x axis indicates the residue position along the sequence, the y axis indicates the intensity of signal

In this study, we fused PseAAC and PsePSSM algorithms to extract the features of each protein sequence in the dataset, and then extract feature vectors of the proteins by 1-D and 2-D wavelet denoising respectively. The specific process is as follows: Firstly, the wavelet decomposition function of two denoising methods both use db8 wavelet, the scale level is 4. Then the high-frequency coefficients are processed by threshold, and the thresholds are selected by wavelet function to get the default threshold. Finally, the signals are reconstructed based on the low-frequency coefficients of the wavelet decomposition and the high-frequency coefficients after the threshold quantization. In order to more intuitively compare the influence of 1-D and 2-D wavelet denoising on the datasets, we pick the protein No. Q13323 in the cytoplasmic protein sequences from the apoptosis protein dataset CL317 as an example, and the results are shown in Figure 10.

It can be seen from Figure 10, Q13323 protein sequence after 1-D and 2-D wavelet denoising, the use of 2-D wavelet denoising can not only effectively removed redundant information in the protein sequence, but also has more advantage over 1-D wavelet denoising in extracting feature signals of the protein itself. From the principle of 1-D and 2-D wavelet denoising, we can see that the frequency band of 1-D wavelet denoising decomposition is not detailed enough. 2-D wavelet denoising can deal with the entire dataset, and the data is denoised from the low-frequency, horizontal, vertical and diagonal scale space. The frequency band of 2-D wavelet denoising decomposition is detailed. We found that 2-D wavelet denoising can effectively improve the feature of each class of apoptosis protein sequences after several comparisons. Therefore, we adopt use the 2-D wavelet denoising method for the apoptosis protein datasets.

### Support vector machine

Support vector machine (SVM) is a machine learning method based on statistical learning theory proposed by Vapnik *et al*. [82]. In recents years, it has been introduced to solve various pattern recognition problems. For the bioinformatics data with high dimensionality, small sample and non-linearity, SVM has excellent learning performance under the principle of structural risk minimization. It is widely used to predict membrane protein types [83], G protein-coupled receptors [84], protein structural classes [55, 85, 86], protein-protein interaction [87], protein subcellular localization [42, 88], protein post-translational modification sites [89, 90] and other protein function prediction research.

For a two-class classification problem, suppose a training set with *n* samples $G=\left\{\left(x_{i},y_{i}\right)|i=1,2,\cdots ,n\right\}\;x_{i}\in R^{d},y_{i}\in \left\{+1,-1\right\},$ where *x_i_* is *d* dimension feature vectors of sample *i*, *y_i_* is the class labels of sample *i*. In order to complete the classification of the two classes of samples, SVM maps the samples of the input vector into a high-dimensional feature space using some nonlinear kernel function, and constructing an optimal separating hyperplane in this space to make the two samples linearly separable.

The kernel function *K*(*x_i_*, *x_j_*) is used to replace the dot product in the optimal classification hyperplane, the decision function is given by:

$$f\left(x\right)=sgn\left\{\sum_{i=1}^{n}\alpha_{i}y_{i}K(x_{i},x_{j})+b\right\}$$ (10)

where *a_i_* denotes Lagrange multiplier, *b* is classification threshold value, *K*(*x_i_*, *x_j_*) is the kernel function. The selection of kernel function is very important in the process of training the support vector. It can effectively overcome the “curse of dimensionality”. The appropriate kernel function can improve the prediction accuracy of the classification model. Generally, four kinds of kernel functions, i.e. linear kernel function, polynomial kernel function, radial basis function (RBF) and sigmoid kernel function, can be available to perform prediction.

SVM is originally designed for two-class classification, and protein subcellular localization prediction is a multi-class classification problem. Currently, there are three main strategies for multi-classification: one-versus-one (OVO), one-versus-rest (OVR) [91] and direct acyclic graph (DAG) [92]. In this paper, we use the OVO strategy. For a *k*-class problem, we need to train k×(k−1)/2 two-class SVM classifiers. Each classifier is used to distinguish which class it belongs to respectively. Votes for each class are counted and the class with the most votes as the predict class. This study used LIBSVM package developed by Chang and Lin [93], which can be freely downloaded from http://www.csie.ntu.edu.tw/~cjlin/libsvm/.

### Performance evaluation and model building

In statistical prediction, four validation tests are often used to evaluate the prediction performance: independent dataset test, jackknife test, self-consistency test and k-fold cross-validation. In this study, jackknife test and self-consistency test method are used to examine the performance of the prediction model.

To evaluate the performance of related predictive methods, we report five standard performance measures, including Sensitivity, Specificity, Matthew's correlation coefficient (MCC), Overall accuracy (OA) and Area Under the ROC Curve (AUC). These measures are defined as follows:

$$Sens=\frac{TP}{TP+FN}$$ (11)

$$Spec=\frac{TN}{TN+FP}$$ (12)

$$MCC=\;\frac{TP\times TN-FP\times FN}{\sqrt{\left(TP+FP\right)\times \left(TP+FN\right)\times \left(TN+FP\right)\times \left(TN+FN\right)}}$$ (13)

$$OA=\frac{TP+TN}{TP+FN+FP+TN}$$ (14)

where *TP* represents the number of true positives, *FB* represents the number of false positives, *TN* represents the number of true negatives, *FN* represents the number of false negatives. In addition, the receiver operating characteristic (ROC) curve can test the generalization performance of the model. The AUC is used as a quantitative indicator of the robustness of the model. In general, the larger the area covered by ROC, the higher the AUC value and the better the generalization performance of the model.

**Figure 11:**
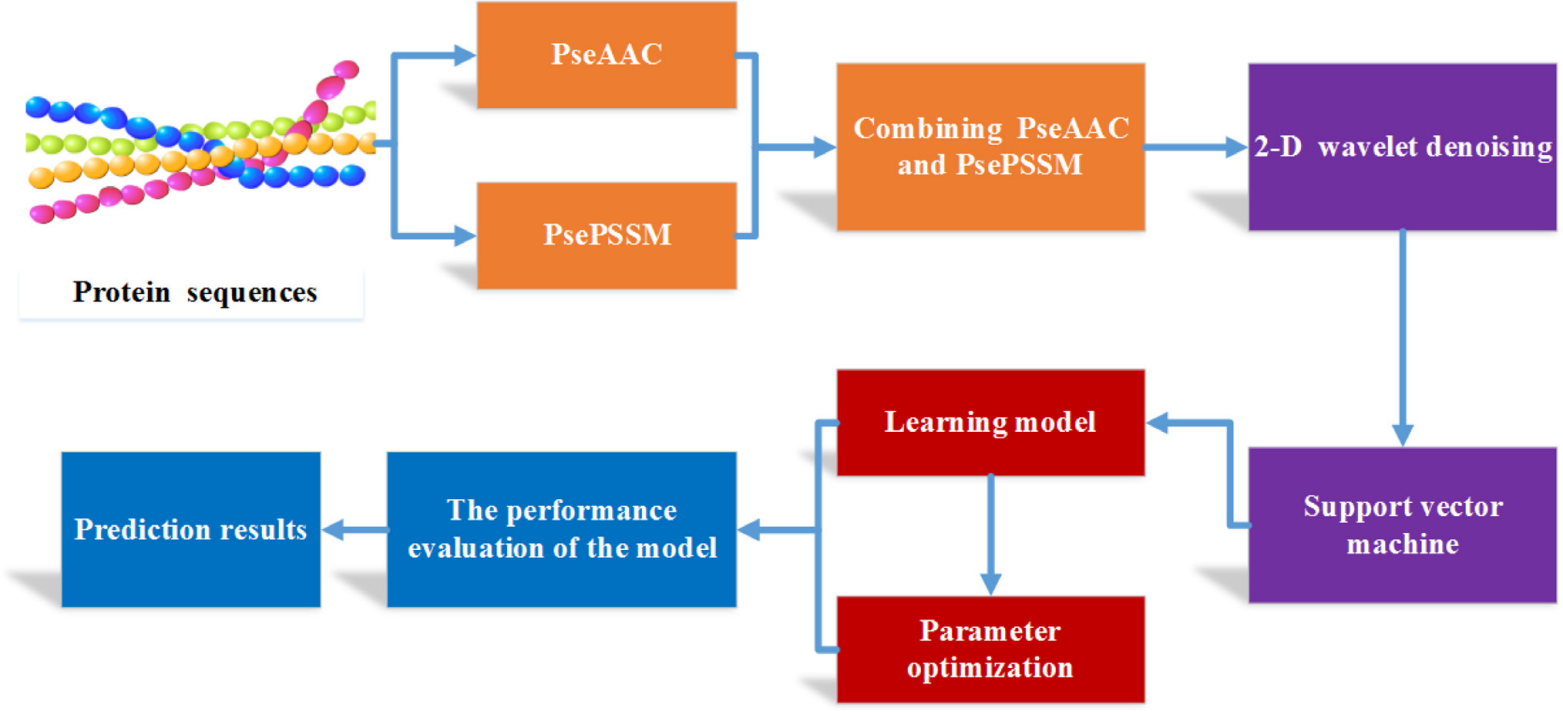
Flow chart of apoptosis protein subcellular localization prediction based on PseAAC-PsePSSM-WD method

For convenience, the method for predicting apoptosis protein subcellular localization in this paper is called PseAAC-PsePSSM-WD, and the general framework is shown in Figure 11. We have implemented it in MATLAB R2014a in windows Server 2012R2 running on a PC with system configuration Intel (R) Xeon (TM) CPU E5-2650 @ 2.30GHz 2.30GHz with 32.0GB of RAM.

The steps of the PseAAC-PsePSSM-WD prediction method are described as follows:

1) Input protein sequences of two different apoptosis datasets CL317 and ZW225, respectively, and the class label corresponding to all kinds of proteins;

2) Use PseAAC online service system developed by Shen and Chou [52] to carry out feature extraction on the protein sequences and generate 20 + λ Bdimension feature vector. The 20 + 20 × ξ dimension feature vector is generated by PsePSSM. Two methods are combined to generate X=317×((20+λ)+(20+20×ξ)) and X=225×((20+λ)+(20+20×ξ)) dimension feature vectors from the CL317 and ZW225 datasets, respectively;

3) Wavelet denoising method is used to denoise the numerical sequences extracted in 2), and the redundant information in the sequence is eliminated;

4) The optimal feature vectors of noise reduction are input to the SVM classifier, and to the protein subcellular localization is predicted by jackknife test;

5) According to the accuracy of prediction, choose the optimal parameters of the model, including the *λ* values and *ξ* values of parameters, wavelet function selection, different decomposition scales and kernel functions in SVM;

6) According to the optimal parameters of the predicted model, *Sens*, *Spec*, MCC and OA are calculated, and the prediction performance of the model is evaluated;

7) Using the model constructed by 1) – 6), the independent testset ZD98 is used to test the prediction model.

## SUPPLEMENTARY MATERIALS TABLES


