# Supplementary material for: Accurate prediction of subcellular location of apoptosis proteins combining Chou’s PseAAC and PsePSSM based on wavelet denoising
Source: Oncotarget. 2017 Nov 21;8(64):107640–65. doi: 10.18632/oncotarget.22585 (PMC5746097; doi:10.18632/oncotarget.22585)
Supplement: Supplementary file 1 [file oncotarget-08-107640-s001.pdf]

Accurate prediction of subcellular location of apoptosis proteins combining Chou's PseAAC and PsePSSM based on wavelet denoising

SUPPLEMENTARY MATERIALS

Supplementary Table 1: Prediction results of subcellular localization of the CL317 dataset by selecting different threshold

Thresholds		Wavelet functions Jackknife test (%)									
		db1	db4	db8	sym3	sym7	coif2	coif4	bior1.1	bior2.4	bior3.3
3	Default(wv)	99.05	98.11	99.05	98.74	98.11	98.11	97.48	99.05	98.42	98.74
	Default(wp)	96.53	98.42	98.42	98.11	98.74	98.42	98.42	96.53	99.05	97.79
	Birge-Massart	99.05	97.16	99.05	97.79	97.79	97.79	98.11	99.05	98.42	98.11
4	Default(wv)	98.74	97.48	99.37	98.11	97.79	98.74	98.42	98.74	98.42	98.74
	Default(wp)	96.53	98.42	98.42	98.11	98.74	98.42	98.42	96.53	98.74	98.42
	Birge-Massart	95.27	98.42	99.05	97.79	98.42	97.79	98.11	95.27	98.74	98.74
5	Default(wv)	98.74	97.79	99.05	98.74	98.42	98.11	98.42	98.74	98.42	98.74
	Default(wp)	96.53	98.42	97.79	98.42	98.74	98.42	98.11	96.53	98.74	98.74
	Birge-Massart	81.07	98.74	98.11	98.74	97.79	97.79	98.11	81.07	98.11	98.42

'wv' means default threshold obtained by wavelet function.
'wp' means default threshold obtained by wavelet packet function.

Supplementary Table 2: Prediction results of subcellular localization of ZW225 apoptosis proteins dataset by selecting different threshold

Thresholds		Wavelet functions Jackknife test (%)									
		db1	db4	db8	sym3	sym7	coif2	coif4	bior1.1	bior2.4	bior3.3
3	Default(wv)	98.67	98.67	100	99.11	99.11	99.56	99.56	98.67	98.22	99.11
	Default(wp)	98.67	97.78	98.22	98.22	99.11	98.67	98.22	98.67	99.11	98.22
	Birge-Massart	97.78	97.33	99.11	98.67	98.67	98.67	97.78	97.78	98.22	97.33
4	Default(wv)	98.22	98.67	100	98.22	98.67	99.11	99.11	98.22	97.78	99.11
	Default(wp)	98.67	98.22	98.67	98.22	98.67	98.67	99.11	98.67	98.67	98.22
	Birge-Massart	89.78	98.22	99.11	98.22	99.11	99.11	98.67	89.78	99.11	98.67
5	Default(wv)	97.78	98.22	99.56	99.11	99.11	98.67	99.56	97.78	97.78	98.67
	Default(wp)	98.67	98.22	98.67	98.22	98.67	99.11	98.67	98.67	98.67	97.78
	Birge-Massart	92.89	98.67	98.67	98.67	98.67	98.67	98.22	92.89	98.22	98.67

'wv' means default threshold obtained by wavelet function;
'wp' means default threshold obtained by wavelet packet function.

Supplementary Table 3: Prediction results of subcellular localization of the CL317 dataset under different classification algorithms

Locations	Classifiers				
	Jackknife test (%)				
	SVM	KNN	RF	Naïve Bayes	DT
Cy	100	99.11	100	88.39	97.32
Me	100	100	98.18	83.64	94.55
Mi	94.12	97.06	91.18	85.29	85.29
Se	100	100	100	100	94.12
Nu	100	100	94.23	84.62	90.38
En	100	97.87	100	95.74	100
OA	99.37	99.05	97.79	88.33	94.64

Supplementary Table 4: Prediction results of subcellular localization of the ZW225 dataset under different classification algorithms

Locations	Classifiers				
	Jackknife test (%)				
	SVM	KNN	RF	Naïve Bayes	DT
Cy	100	100	100	100	100
Me	100	98.88	98.88	85.39	97.75
Mi	100	76.00	96.00	96.00	92.00
Nu	100	100.00	100.00	100.00	100.00
OA	100	96.89	99.11	93.78	98.22
